# 2020 WSES guidelines for the detection and management of bile duct injury during cholecystectomy

**DOI:** 10.1186/s13017-021-00369-w

**Published:** 2021-06-10

**Authors:** Nicola de’Angelis, Fausto Catena, Riccardo Memeo, Federico Coccolini, Aleix Martínez-Pérez, Oreste M. Romeo, Belinda De Simone, Salomone Di Saverio, Raffaele Brustia, Rami Rhaiem, Tullio Piardi, Maria Conticchio, Francesco Marchegiani, Nassiba Beghdadi, Fikri M. Abu-Zidan, Ruslan Alikhanov, Marc-Antoine Allard, Niccolò Allievi, Giuliana Amaddeo, Luca Ansaloni, Roland Andersson, Enrico Andolfi, Mohammad Azfar, Miklosh Bala, Amine Benkabbou, Offir Ben-Ishay, Giorgio Bianchi, Walter L. Biffl, Francesco Brunetti, Maria Clotilde Carra, Daniel Casanova, Valerio Celentano, Marco Ceresoli, Osvaldo Chiara, Stefania Cimbanassi, Roberto Bini, Raul Coimbra, Gian Luigi de’Angelis, Francesco Decembrino, Andrea De Palma, Philip R. de Reuver, Carlos Domingo, Christian Cotsoglou, Alessandro Ferrero, Gustavo P. Fraga, Federica Gaiani, Federico Gheza, Angela Gurrado, Ewen Harrison, Angel Henriquez, Stefan Hofmeyr, Roberta Iadarola, Jeffry L. Kashuk, Reza Kianmanesh, Andrew W. Kirkpatrick, Yoram Kluger, Filippo Landi, Serena Langella, Real Lapointe, Bertrand Le Roy, Alain Luciani, Fernando Machado, Umberto Maggi, Ronald V. Maier, Alain Chichom Mefire, Kazuhiro Hiramatsu, Carlos Ordoñez, Franca Patrizi, Manuel Planells, Andrew B. Peitzman, Juan Pekolj, Fabiano Perdigao, Bruno M. Pereira, Patrick Pessaux, Michele Pisano, Juan Carlos Puyana, Sandro Rizoli, Luca Portigliotti, Raffaele Romito, Boris Sakakushev, Behnam Sanei, Olivier Scatton, Mario Serradilla-Martin, Anne-Sophie Schneck, Mohammed Lamine Sissoko, Iradj Sobhani, Richard P. ten Broek, Mario Testini, Roberto Valinas, Giorgos Veloudis, Giulio Cesare Vitali, Dieter Weber, Luigi Zorcolo, Felice Giuliante, Paschalis Gavriilidis, David Fuks, Daniele Sommacale

**Affiliations:** 1 Unit of Minimally Invasive and Robotic Digestive Surgery, General Regional Hospital “F. Miulli”, Strada Prov. 127 Acquaviva – Santeramo Km. 4, 70021 Acquaviva delle Fonti BA, Bari, Italy; 2 Unit of Digestive, Hepatobiliary and Pancreatic Surgery, CARE Department, Henri Mondor University Hospital (AP-HP), and Faculty of Medicine, University of Paris Est, UPEC, Creteil, France; 3 Department of Emergency and Trauma Surgery of the University Hospital of Parma, Parma, Italy; 4 Department of Hepato-Pancreatic-Biliary Surgery, General Regional Hospital “F. Miulli”, Acquaviva delle Fonti, Bari, Italy; 5 General, Emergency and Trauma Department, Pisa University Hospital, Pisa, Italy; 6 Department of General and Digestive Surgery, Hospital Universitario Doctor Peset, Valencia, Spain; 7 Trauma, Burn, and Surgical Care Program, Bronson Methodist Hospital, Kalamazoo, Michigan USA; 8 Service de Chirurgie Générale, Digestive, et Métabolique, Centre hospitalier de Poissy/Saint Germain en Laye, Saint Germain en Laye, France; 9 Department of Surgery, Cambridge University Hospital, NHS Foundation Trust, Cambridge, UK; 10 Department of HBP and Digestive Oncologic Surgery, Robert Debré University Hospital, Reims, France; 11 Department of Surgery, HPB Unit, Troyes Hospital, Troyes, France; 12 Department of Surgical, Oncological and Gastroenterological Sciences, University of Padua, Padua, Italy; 13 Department of Surgery, College of Medicine and Health Sciences, UAE University, Al-Ain, United Arab Emirates; 14 Department of Hepato-Pancreato-Biliary Surgery, Moscow Clinical Scientific Center, Shosse Enthusiastov, 86, 111123 Moscow, Russia; 15 Centre Hepatobiliaire, Paul Brousse Hospital, Villejuif, France; 16 1st Surgical Unit, Department of Emergency, Papa Giovanni Hospital XXIII, Bergamo, Italy; 17 Service d’Hepatologie, APHP, Henri Mondor University Hospital, Creteil, and Faculty of Medicine, University of Paris Est, UPEC, Creteil, France; 18 General Surgery, San Matteo University Hospital, Pavia, Italy; 19 Department of Surgery, Linkonping University, Linkoping, Sweden; 20 Department of Surgery, Division of General Surgery, San Donato Hospital, 52100 Arezzo, Italy; 21 Department of Surgery, Al Rahba Hospital, Abu Dhabi, UAE; 22 Trauma and Acute Care Surgery Unit, Hadassah Hebrew University Medical Center, Jerusalem, Israel; 23 Surgical Oncology Department, National Institute of Oncology, Mohammed V University in Rabat, Rabat, Morocco; 24 Department of General Surgery, Rambam Healthcare Campus, Haifa, Israel; 25 Division of Trauma and Acute Care Surgery, Scripps Memorial Hospital La Jolla, La Jolla, California USA; 26 Rothschild Hospital, AP-HP, Paris, and Université de Paris, Paris, France; 27 Hospital Universitario Marqués de Valdecilla, University of Cantabria, Santander, Spain; 28 Colorectal Unit, Chelsea and Westminster Hospital, NHS Foundation Trust, London, UK; 29 Emergency and General Surgery Department, University of Milan Bicocca, Milan, Italy; 30 General Surgery and Trauma Team, ASST Niguarda Milano, University of Milano, Milan, Italy; 31 Riverside University Health System Medical Center, Comparative Effectiveness and Clinical Outcomes Research Center – CECORC and Loma Linda University School of Medicine, Loma Linda, USA; 32 Gastroenterology and Endoscopy Unit, Department of Medicine and Surgery, University of Parma, Parma, Italy; 33 Gastroenterology and Endoscopy Unit, General Regional Hospital “F. Miulli”, Acquaviva delle Fonti, Bari, Italy; 34 Department of Surgery, Radboud University Medical Centre Nijmegen, Nijmegen, The Netherlands; 35 General Surgery Department, ASST-Vimercat, 20871 Vimercate, Italy; 36 Department of General and Oncological Surgery, Azienda Ospedaliera Ordine Mauriziano “Umberto I”, Turin, Italy; 37 Division of Trauma Surgery, Department of Surgery, School of Medical Sciences, University of Campinas (Unicamp), Campinas, SP Brazil; 38 Department of Clinical and Experimental Sciences, University of Brescia, Brescia, Italy; 39 Unit of General Surgery “V. Bonomo”, Department of Biomedical Sciences and Human Oncology, University of Bari “Aldo Moro”, Bari, Italy; 40 Department of Clinical Surgery and Centre for Medical Informatics, Usher Institute, University of Edinburgh, Little France Crescent, Edinburgh, UK; 41 Centro Médico Laparoscópic, San Salvador, El Salvador; 42 Division of Surgery, Surgical Gastroenterology Unit, Tygerberg Academic Hospital, University of Stellenbosch Faculty of Medicine and Health Sciences, Stellenbosch, South Africa; 43 Department of Surgery, Tel Aviv University, Sackler School of Medicine, Tel Aviv, Israel; 44 Department of Surgery, Critical Care Medicine and the Regional Trauma Service, Foothills Medical Center, Calgari, Alberta Canada; 45 Department of HPB and Transplant Surgery, Hospital Clínic, Universidad de Barcelona, Barcelona, Spain; 46 Department of HBP Surgery and Liver Transplantation, Department of Surgery, Centre Hospitalier de l’Université de Montreal, Montreal, QC Canada; 47 Department of Digestive Surgery, University Hospital of Saint-Etienne, Saint-Priest-en-Jarez, France; 48 Unit of Radiology, Henri Mondor University Hospital (AP-HP), Creteil, and Faculty of Medicine, University of Paris Est, UPEC, Creteil, France; 49 Department of Emergency Surgery, Hospital de Clínicas, School of Medicine UDELAR, Montevideo, Uruguay; 50 General Surgery and Liver Transplantation Unit, Fondazione IRCCS Ca’Granda, Ospedale Maggiore Policlinico di Milano, Milan, Italy; 51 Department of Surgery, University of Washington, Seattle, WA USA; 52 Department of Surgery and Obstetrics/Gynecologic, Regional Hospital, Limbe, Cameroon; 53 Department of General Surgery, Toyohashi Municipal Hospital, Toyohashi, Aichi Japan; 54 Division of Trauma and Acute Care Surgery, Department of Surgery, Fundacion Valle del Lili, Universidad del Valle Cali, Cali, Colombia; 55 Unit of Gastroenterology and Endoscopy, Maggiore Hospital, Bologna, Italy; 56 Department of Surgery, UPMC, University of Pittsburg, School of Medicine, Pittsburg, USA; 57 General Surgery, Liver Transplant Unit, Hospital Italiano de Buenos Aires, Buenos Aires, Argentina; 58 Liver Transplant Unit, APHP, Unité de Chirurgie Hépatobiliaire et Transplantation hépatique, Hôpital Pitié Salpêtrière, Paris, France; 59 Hepatobiliary and Pancreatic Surgical Unit, Visceral and Digestive Surgery, IHU mix-surg, Institute for Minimally Invasive Image-Guided Surgery, University of Strasbourg, Strasbourg, France; 60 Trauma & Acute Care Surgery – Global Health, University of Pittsburgh, Pittsburgh, USA; 61 Trauma and Acute Care Service, St Michael’s Hospital, Toronto, ON Canada; 62 Chirurgia Epato-Gastro-Pancreatica, Azienda Ospedaliera-Universitaria Maggiore della Carità, Novara, Italy; 63 General Surgery Department, Medical University, University Hospital St George, Plovdiv, Bulgaria; 64 Department of Surgery, Isfahan University of Medical Sciences, Isfahan, Iran; 65 Instituto de Investigación Sanitaria Aragón, Department of Surgery, Hospital Universitario Miguel Servet, Zaragoza, Spain; 66 Digestive Surgery Unit, Centre Hospitalier Universitaire de Guadeloupe, Pointe-À-Pitre, Les Avymes, Guadeloupe France; 67 Service de Chirurgie, Hôpital National Blaise Compaoré de Ouagadougou, Ouagadougou, Burkina Faso; 68 Department of Gastroenterology and Digestive Endoscopy, Henri Mondor Hospital, AP-HP, Creteil, and Faculty of Medicine, University of Paris Est, UPEC, Creteil, France; 69 Department of Surgery “F”, Faculty of Medicine, Clinic Hospital “Dr. Manuel Quintela”, Montevideo, Uruguay; 70 Veterans Fund Army Hospital, Athens, Greece; 71 Division of Transplantation, Department of Surgery, Geneva University Hospitals, Geneva, Switzerland; 72 Department of Trauma Surgery, Royal Perth Hospital, Perth, Australia; 73 Department of Surgery, Colorectal Surgery Unit, University of Cagliari, Cagliari, Italy; 74 Hepatobiliary Surgery Unit, Foundation “Policlinico Universitario A. Gemelli”, IRCCS, Rome, Italy; 75 Division of Gastrointestinal and HBP Surgery, Imperial College HealthCare, NHS Trust, Hammersmith Hospital, London, UK; 76 Institut Mutualiste Montsouris, Paris, France

**Keywords:** Laparoscopic cholecystectomy, Biliary duct injury, Magnetic resonance imaging, Antibiotic therapy, Computed tomography, Guidelines

## Abstract

Bile duct injury (BDI) is a dangerous complication of cholecystectomy, with significant postoperative sequelae for the patient in terms of morbidity, mortality, and long-term quality of life. BDIs have an estimated incidence of 0.4–1.5%, but considering the number of cholecystectomies performed worldwide, mostly by laparoscopy, surgeons must be prepared to manage this surgical challenge. Most BDIs are recognized either during the procedure or in the immediate postoperative period. However, some BDIs may be discovered later during the postoperative period, and this may translate to delayed or inappropriate treatments. Providing a specific diagnosis and a precise description of the BDI will expedite the decision-making process and increase the chance of treatment success. Subsequently, the choice and timing of the appropriate reconstructive strategy have a critical role in long-term prognosis. Currently, a wide spectrum of multidisciplinary interventions with different degrees of invasiveness is indicated for BDI management. These World Society of Emergency Surgery (WSES) guidelines have been produced following an exhaustive review of the current literature and an international expert panel discussion with the aim of providing evidence-based recommendations to facilitate and standardize the detection and management of BDIs during cholecystectomy. In particular, the 2020 WSES guidelines cover the following key aspects: (1) strategies to minimize the risk of BDI during cholecystectomy; (2) BDI rates in general surgery units and review of surgical practice; (3) how to classify, stage, and report BDI once detected; (4) how to manage an intraoperatively detected BDI; (5) indications for antibiotic treatment; (6) indications for clinical, biochemical, and imaging investigations for suspected BDI; and (7) how to manage a postoperatively detected BDI.

**Supplementary Information:**

The online version contains supplementary material available at 10.1186/s13017-021-00369-w.

## Background

The World Society of Emergency Surgery (WSES) was founded in 2007 with the mission of promoting training and continuing medical education in emergency general surgery and trauma. Since its establishment, the WSES has launched and curated several clinical guidelines for specific topics related to emergency and trauma surgery, which are regularly updated to provide evidence-based guidance to emergency surgeons in their daily practice [1–3]. From this perspective, the present manuscript describes the international work conducted by WSES members to build consensus guidelines for the detection and management of one of the most severe complications of cholecystectomy, namely, bile duct injury (BDI).

Laparoscopic cholecystectomy (LC) is the gold standard operation for patients with gallstone disease and represents one of the most common routine interventions performed worldwide in both elective and emergency settings [4, 5]. Bile duct injuries (BDIs) are dangerous complications of cholecystectomy, occurring more often since the introduction and widespread adoption of laparoscopy (0.4–1.5% of cases) compared to open cholecystectomy (0.2–0.3% of cases) [4, 6–9]. Since early reports, the frequency of BDIs during LC has been progressively decreasing. However, the injuries seen currently tend to be more severe, with the most severe biliary and hepatic artery or portal vein injuries often occurring after conversion from laparoscopy to open cholecystectomy [5, 10].

BDIs are a surgical challenge associated with significant postoperative sequelae for the patient in terms of morbidity, mortality (up to 3.5%), and long-term quality of life [11–13]. Injuries of the bile duct system occurring during cholecystectomy are complex and require prompt identification and management. Visualization of BDIs might be hindered by accompanying vascular injuries, particularly in the branches of the right hepatic artery. Failed attempts to repair BDIs can result in longitudinal strictures of the common bile duct [7, 11, 14–16]. Most BDIs are recognized either during the procedure or in the immediate postoperative period, with the two most frequent scenarios being the occurrence of a bile leak or bile duct obstruction [11, 17]. However, some BDIs may be discovered later in the postoperative period, and this often translates to delayed or inappropriate treatments, especially when BDI patients need to be referred from a secondary hospital to a tertiary care center for definitive management. Providing a specific diagnosis and a precise description of the BDI facilitates the decision-making process and increases the chance of treatment success [9, 18]. In delayed cases, the choice and timing of the appropriate reconstructive procedure have a critical role in long-term prognosis.

Currently, there is a wide spectrum of interventions used in the management of BDI with different degrees of invasiveness, ranging from computed tomography (CT)-guided drainage to various endoscopic and surgical techniques. With such a variety of interdisciplinary options available and the need to act promptly, close cooperation between gastroenterologists, radiologists, and surgeons is of upmost importance [4, 9, 17–20].

The present WSES guidelines aim to facilitate efficient interdisciplinary cooperation, providing evidence-based recommendations for the prevention, detection, and management of BDIs during cholecystectomy. The process was initiated in April 2019 and was structured around 7 key questions that were addressed by comprehensive literature reviews conducted by 7 groups of international multidisciplinary experts. The worldwide participation in these WSES guidelines was deemed essential to capture the experience and practice of different realities in multiple countries, beyond the evidence in the literature, and to ultimately propose clinical guidelines that can contribute to standardizing BDI treatments and research objectives in the future. The present guidelines apply for all cholecystectomy-related BDI regardless of the surgical approach. However, being LC the gold standard with the great majority of the literature referring to this procedure, it will be the most frequently considered in the following recommendations.

## Guideline scope and methods

In April 2019, the President and the scientific committee of the WSES appointed three experts (Fausto Catena, Nicola de’Angelis, and Daniele Sommacale) to establish the project committee and determine the organization of an international multidisciplinary expert panel to develop the WSES Guidelines for the detection and management of BDIs. Briefly, the development of the WSES guidelines was structured in two steps: a synthesis of the current literature and a consensus conference held during the 7^th^ WSES World Congress.

In the first step, the project committee identified 7 key questions regarding BDIs to be addressed by a thorough analysis of the available literature. Seven groups of experts, including surgeons, anesthesiologists, gastroenterologists, hepatologists, and radiologists, were identified (Table 1). For each working group, a leader and co-leader(s) were designated as responsible for coordinating the work of the group’s experts and providing a summary document that aligned the group’s recommendations.

**Table 1:** The 7 key questions and the working groups of experts who contributed to the WSES guidelines on biliary duct injury (BDI) detection and management

**Question n° 1: What are the general recommendations to minimize the risk of BDI during laparoscopic cholecystectomy in elective and emergency settings?**		
**Team leader** Federico Coccolini	**Co-leads** Federico Gheza and Andrea De Palma	**Working group members** Miklosh Bala, Ofir Ben-Ishay, Marco Ceresoli, Stefania Cimbanassi, Philip de Reuver, Bertrand Le Roy, Chichom Mefire, Andrew Kirkpatrick, Carlos Ordoñez, Richard ten Broek and Dieter Weber
**Question n° 2: What are the reported BDI rates during LC in emergency and elective settings and when should a surgical team review its current practice to improve the standards of care?**		
**Team leader** Aleix Martinez-Perez	**Co-leads** Salomone Di Saverio	**Working group members** Luca Ansaloni, Daniel Casanova, David Fuks, Carlos Domingo, Manuel Planells, Yoram Kluger, Filippo Landi, Andrew B. Peitzman, Sandro Rizoli and Mario Serradilla-Martin
**Question n° 3: Which classifications of BDI should be adopted and what is the minimum required information that the surgeon must report after diagnosing BDI during laparoscopic cholecystectomy?**		
**Team leader** Nicola de’Angelis	**Co-leads** Nassiba Beghdadi	**Working group members** Fikri M. Abu-Zidan, Marc-Antoine Allard, Francesco Brunetti, Maria Clotilde Carra, Valerio Celentano, Christian Cotsoglou, Federica Gaiani, Reza Kianmanesh, Real Lapointe, Bruno M. Pereira, Luca Portigliotti and Giorgos Veloudis
**Question n° 4: What are the surgical management strategies and timing for intraoperatively diagnosed BDI?**		
**Team leader** Daniele Sommacale	**Co-leads** Raffaele Brustia	**Working group members** Ruslan Alikhanov, Alessandro Ferrero, Felice Giuliante, Stefan Hofmeyr, Mohammed Lamine Sissoko, Serena Langella, Kazuhiro Niramatsu, Juan Pekolj, Fabiano Perdigao, Behnam Sanei, Olivier Scatton, Boris Sakakushev and Roberto Valinas
**Question n° 5: What is the recommended type and duration of antibiotic regimen in cases of BDI?**		
**Team leader** Oreste M. Romeo	**Co-leads** Tullio Piardi and Rami Rhaiem	**Working group members** Niccolò Allievi, Roland Andersson, Enrico Andolfi, Walter Biffl, Raul Coimbra, Gustavo Fraga, Angela Gurrado, Michele Pisano, Raffaele Romito, Anne-Sophie Schnek and Giulio Vitali
**Question n° 6: Which are the clinical, biochemical, and imaging investigations required for the postoperative diagnosis of BDI?**		
**Team leader** Fausto Catena	**Co-leads** Belinda de Simone	**Working group members** Giuliana Amaddeo, Osvaldo Chiara, Roberto Bini, Gian Luigi de’Angelis, Francesco Decembrino, Federica Gaiani, Roberta Iadarola, Alain Luciani, Ronald V. Maier, Franca Patrizi, Juan Carlos Puyana, Iradj Sobhani, Mario Testini and Luigi Zorcolo
**Question n° 7: What are the surgical management strategies and timing for postoperatively diagnosed BDI?**		
**Team leader** Riccardo Memeo	**Co-leads** Maria Conticchio and Francesco Marchegiani	**Working group members** Mohammad Azfar, Amine Benkabbou, Raffaele Brustia, Salomone Di Saverio, Paschalis Gavriilidis, Ewen Harrison, Umberto Maggi, Angel Henriquez, Stefan Hofmeyr, Jeffry L Kashuk, Fernando Machado, Patrick Pessaux, Behnam Sanei and Daniele Sommacale

The literature evaluation was conducted by performing bibliographic searches related to the 7 key questions using a systematic approach and exploring different electronic databases, including PubMed and EMBASE. There was no date or language restriction. Within each group, a scientific discussion ensued via email and/or videoconference and a synthesis document based on literature evidence, clinical experience, and expert discussion were developed. Experts were instructed to formulate statements and recommendations, as they did for previous WSES guidelines [2, 21, 22], and assess the level of evidence and the strength of the recommendations according to the AGREE II requirements and adopting the Grading of Recommendations Assessment, Development and Evaluation (GRADE) criteria (https://www.gradeworkinggroup.org/) [23, 24]. The quality of evidence was graded as “High,” “Moderate,” “Low,” or “Very low,” whereas the strength of a recommendation was indicated as either “Strong” or “Weak.” Statements and recommendations were reviewed by the project committee to create a comprehensive draft version of the guidelines, including all 7 key questions to be available prior to the consensus conference.

The consensus conference was planned during the 7^th^ WSES World Congress that was initially scheduled to take place in Milano in June 2020. Due to the COVID-19 pandemic, the event was rescheduled to occur on the 16^th^–19^th^ of November 2020 using a virtual format. During the conference, a representative of the project committee presented the summary documents of the working groups and detailed the statements and recommendations, the supporting literature, and the level and strength of the supporting evidence.

The revised statements, their level of evidence, and their recommendation grades are presented below. Please note that the WSES guidelines must be considered as an adjunctive tool in the decision-making process regarding the management of BDIs; they are not intended to substitute a provider’s clinical judgment regarding an individual patient or specific clinical situation, and they may need to be adapted to be consistent with the medical team’s experience and the available local resources.

### BDI key questions

**Table:** 

**Q1. What are the general recommendations to minimize the risk of BDI during laparoscopic cholecystectomy in elective and emergency settings?**	
**Statements:**	
1.1. The use of the CVS during LC (achieving all 3 components) is the recommended approach to minimize the risk of BDIs. *Strong recommendation, low quality of evidence (GRADE 1C)* 1.2. If the CVS is not achievable during a difficult LC, a bailout procedure, such as STC, should be considered. *Strong recommendation, moderate quality of evidence (GRADE 1B)* 1.3. Conversion to open surgery may be considered during a difficult LC whenever the operating surgeon cannot manage the procedure laparoscopically. However, there is insufficient evidence to support conversion to open surgery as a strategy to avoid or reduce the risk of BDI in difficult LCs. *Weak recommendation, moderate quality of evidence (GRADE 2B)* 1.4. Intraoperative IOC is useful to recognize bile duct anatomy and choledocholithiasis in cases of intraoperative suspicion of BDI, misunderstanding of biliary anatomy, or inability to see the CVS, but routine use to reduce the BDI rate is not yet recommended. *Weak recommendation, high quality of evidence (GRADE 2A)* 1.5. Intraoperative ICG-C is a promising noninvasive tool to recognize bile duct anatomy and vascular structures, but routine use to reduce the BDI rate is not yet recommended. *Weak recommendation, low quality of evidence (GRADE 2C).* 1.6. In patients presenting with AC, the optimal timing for cholecystectomy is within 48 h, and no more than 10 days from symptom appearance. *Strong recommendation, good quality of evidence* (*GRADE 1A*) 1.7. In patients with at-risk conditions (e.g., scleroatrophic cholecystitis, Mirizzi syndrome), an exhaustive preoperative work-up prior cholecystectomy is mandatory in order to discuss and balance the risks/benefits ratio of the procedure. *Weak recommendation, low quality of evidence (GRADE 2C)*	

### Literature review

Due to the potentially severe consequences of BDIs, all efforts should be made to minimize the risk of occurrence in both elective and emergency cholecystectomies. Optimal strategies for the prevention of BDI include technical and procedural considerations that must be adapted based on anatomical factors, the patient’s clinical status, disease factors, and the surgeon’s experience [5]. An exhaustive preoperative work-up prior to cholecystectomy is mandatory in order to detect at-risk conditions (e.g., scleroatrophic cholecystitis, Mirizzi syndrome [25–27]), choose the best surgical approach, and discuss the risks/benefits ratio of the procedure.

#### Critical view of safety

LC is the preferable approach also for AC and is associated with lower mortality and morbidity rates [28–34]. The risk of conversion to open surgery appears to be higher with male sex, age > 60 years, obesity, cirrhosis, previous upper abdominal surgery, presence of comorbidity, large bile stones, fever, elevated serum bilirubin levels, gangrenous cholecystitis, severe acute, and chronic cholecystitis, contracted gallbladder on imaging, duration of complaints > 48 h, and emergency LC [35–37]. Conversion to open surgery may be considered for patient safety if the operating surgeon cannot manage a difficult LC; however, there is no evidence to support that conversion to open per se will avoid or reduce the risk of BDI [5, 38, 39]. The critical view of safety (CVS) technique was introduced in 1995 to guarantee the safest approach to LC by promoting the recognition of gallbladder elements, particularly the hepatocystic triangle (composed of the cystic duct, common bile duct, and liver) [40, 41], a crucial step to reduce the risk of BDI associated with mistakes in visual perception. The literature has demonstrated that when the CVS is identified, the risk of iatrogenic intraoperative complications is minimized [42–44]. Thus, routine use of CVS is recommended over other techniques, such as the infundibular approach [5, 42, 45, 46]. However, achieving a complete CVS is easily obtained in only 50% of cases. The component most commonly incomplete is clearance of the lower third of the gallbladder from the liver bed, and CVS cannot always be applied if the hepatocystic angle is affected by advanced inflammation or contracting fibrosis due to preceding episodes of inflammation.

It has been reported that injuries of the common bile duct are more common during the early learning curve in laparoscopic cholecystectomy [47]. Thus, the use of CVS could be of greater importance for trainees and residents; in this scenario, the trainee or resident must secure the CVS, and the supervising surgeon must confirm the CVS before the cystic duct and cystic artery are ligated.

#### Bailout procedures

Whenever a CVS cannot be achieved and the biliary anatomy cannot be clearly defined, alternative techniques such as the “fundus-first (top-down)” approach or subtotal cholecystectomy (STC) should be considered [5, 48]. Several studies have shown how the “fundus-first” technique is associated with reduced rates conversion rate and iatrogenic complications (including BDIs) during difficult operations, such as in cases of severe AC [49–52], although the risk of vascular and biliary injuries cannot be completely eliminated [10, 53]. It is essential to recognize approaching areas of danger during LC and, in response, stop dissection and change to a bailout procedure (STC or cholecystostomy) to minimize the need for conversion and to reduce the risk of BDI [48, 54–56]. However, STC is associated with significantly more surgical site infections, a need for re-interventions, and a longer hospital stay than total cholecystectomy [57]. STC showed advantages over a converted cholecystectomy in which conversion will not solve the difficulty of an inflamed hepatocystic triangle [58].

#### Intraoperative biliary imaging

Intraoperative cholangiography (IOC) is an imaging technique that may be used during LC to recognize choledocholithiasis and define the biliary anatomy [59]. However, its routine use is not currently advisable since it is not associated with a significant reduction in rates of complications and BDIs during LC [60, 61]. Indeed, BDI may also occur after IOC because of misinterpretation of the IOC findings. IOC may be recommended in cases of intraoperative suspicion of BDI, misunderstanding of the biliary anatomy, or even inability to see the CVS, as well as in patients with AC or a history of AC, for whom intraoperative imaging, although associated with longer operative time, could be of greatest benefit [5]. Importantly, identification of a BDI using IOC can lead to earlier diagnosis and treatment.

Alternatively*,* the use of indocyanine green fluorescence cholangiography (ICG-C) [62] as an intravenous infusion before surgery can be a useful technique to visualize the structures of the biliary tree, particularly the cystic duct, without the need for X-ray imaging. The usefulness of ICG-C to prevent BDIs has been suggested in several studies and has also proven useful for acute and chronic gallbladder diseases and in those situations in which IOCs cannot be used [63–65].

#### Optimal timing of LC for acute cholecystitis

Systematic reviews analyzing data from RCTs [66] and population-based studies [67, 68] showed higher BDI rates in acute conditions, supporting the hypothesis of increasing BDI risk with increasing severity of local inflammation, such as in the case of acute cholecystitis (AC) [67]. Different time frames for operating on patients presenting with symptomatic AC have been proposed, ranging from no more than 72 h up to 10 days. Further delays are associated with disease progression, and despite medical treatments, unfavorable conditions for safe surgical interventions exist [39]. Indeed, an increase in the complication rate and need of conversion to open cholecystectomy has been reported when the time from symptom appearance to surgery was prolonged [69–71], with the latest timepoint to safely operate on AC patients being 10 days from symptoms appearance [39].

**Table:** 

**Q2. What are the reported BDI rates during LC in emergency and elective settings and when should a surgical team review its current practice to improve the standards of care?**	
**Statement:**	
2.1. Based on large nationwide databases and systematic review of the literature, major BDIs occur in 0.1% of elective LC and 0.3% of emergency LC. If considering all types of BDIs, rates are 0.4% and 0.8% for elective and emergency settings, respectively. When a surgical team experiences an increased rate of BDIs, a careful review of the current practice is mandatory to critically analyze the possible causes and implement educational, training, and technical solutions to improve the standards of care. *Strong recommendation, low quality of evidence (GRADE 1C)*	

### Literature review

Given the number of LCs performed worldwide, thousands of patients per year will experience BDIs with severe and long-term implications for their health. Moreover, BDIs can have a substantial impact on the surgeon’s mental status and reputation and can constitute a non-negligible financial burden for healthcare systems [11].

The goal of any general surgery unit should ideally be a 0% BDI rate, but this is rarely observed in real-life practice. A nationwide database and worldwide experience are necessary to describe the overall incidence of BDIs in elective and emergency settings. Whenever increased rates are experienced locally, the surgical team should carefully review the current practice, critically analyze the possible causes, and implement educational, training, and technical solutions to improve the standards of care.

Epidemiological data are useful for clinicians, surgeons, and healthcare systems to measure surgical outcomes and performance (for monitoring or audit purposes); for patients to weigh the surgical risks; and for researchers to compare and interpret their findings [24]. However, assessing the true frequency of BDIs remains challenging. The main problem is related to the sample size needed to observe BDIs and eventually detect significant changes over time [72].

A systematic literature search limited to articles published between 2011 and 2020 identified 16 studies analyzing 14 different databases from 5 different countries [12, 73–87]. The rates of BDIs during LC differed significantly depending on the population investigated, the criteria used in each study, and the definition of BDI.

Based on the Swedish National Quality Registry of Gallstone Surgery and Endoscopic Retrograde Cholangiopancreatography (GallRiks) established in 2005, Tornqvist et al .[78] analyzed 51,041 cholecystectomies and reported an overall BDI rate of 1.5% according to the Hannover classification system, which includes bile duct leaks [17]. The BDI rates during LC and open cholecystectomy were 1.3% and 2.8%, respectively. AC and emergency admissions were associated with BDI rates of 1.9% and 1.8%, respectively.

A more recent article by Pucher et al. provided an extensive literature review of 151 studies accounting for a total of 505,292 patients undergoing LC [87]. The authors selected only studies including at least 100 patients and excluded those explicitly describing early case experiences or learning curves to ensure representativeness of an established surgical practice. Pooled data analyses (based on 70% of the included studies corresponding to 60% of patients) showed an overall BDI rate ranging from 0.32 to 0.52%. Sixty-five studies differentiated between major and minor injuries and showed a prevalence of 0.28% for major injuries and 0.46% for bile leaks (overall 0.74%) [87].

Several studies defined BDI as the need for further reconstructive surgery (i.e., bilioenteric anastomosis), and they reported a reconstructive surgery rate range between 0.04 and 0.3% [73–77]. When considering only the need for any type of surgical repair of the common bile duct, the rate ranged between 0.06% and 0.31% [73, 76, 82, 83, 85, 88].

The California Cholecystectomy Group analyzed 711,454 cholecystectomies (of which 95% were LCs) from the California Office of Statewide Health Planning and Development (COSHPD) database from 2005 to 2014. They found a bile leak rate of 0.5%, defined by the need for isolated endoscopic retrograde cholangiopancreatography (ERCP) or percutaneous transhepatic cholangiography (PTC) within 4 weeks after cholecystectomy [12]. Patients who underwent choledocho-enterostomy, common bile duct suture, hepatectomy, liver transplantation, more than 1 ERCP within a year, or 1 or more PTCs between 4 weeks and 1 year were considered to have a BDI. The rate of these major BDIs was 0.22%, and together, they accounted for 0.72% of patients requiring any ERCP, PTC, or surgical procedure after LC [12].

A higher incidence of BDIs can be expected in cases of inflammation (acute or chronic) [75, 78–80] or emergency cholecystectomy [75, 78, 79]. Based on the GallRiks database, patients with AC at the time of surgery or with a positive history of AC are at higher risk of BDI (odds ratios, ORs: 1.23 and 1.34, respectively), which can be reduced by performing IOC [78]. Mangieri et al. analyzed 217,774 LCs in the NSQIP database, 67% of which presented with AC. They found a small yet significantly higher incidence rate of BDIs in AC (0.21% vs. 0.18%) [84]. In a nationwide study of 572,223 LCs conducted in England, only a very small difference was reported concerning the need for reconstructive biliary surgery between patients presenting with AC on admission and patients operated on in the elective setting (0.09% vs. 0.11%) [74]. Other studies found no differences in biliary adverse events between LC patients with or without AC [66, 87, 89] or between elective and emergency procedures (0.3%) [81].

**Table:** 

**Q3. Which classifications of BDI should be adopted, and what is the minimum required information that the surgeon must report after diagnosing BDI during LC?**	
**Statements:**	
3.1. We recommend knowing Strasberg’s classification, which remains the most commonly used classification for BDIs, and the ATOM classification, which represents the most recent and complete classification; the implementation of the ATOM classification should be promoted in the near future. *Strong recommendation, low quality of evidence (GRADE 1C)* 3.2. The ideal operative report must maximize the amount of intraoperative detail given to describe the BDI. The following should minimally be included: 1. The clinical context and indication for cholecystectomy 2. Intraoperative findings 3. The anatomical landmarks of the critical view of safety [66, 73, 90] 4. Any anatomical variation of the biliary tract [88, 89] 5. Cholangiography findings (if performed) [66, 81] 6. Operative data (e.g., operative time, blood loss, energy device used for dissection, need for conversion) 7. Drawing of the BDI with biliary drain placement (if used) 8. Videotape of the procedure (whenever available). *Strong recommendation, low quality of evidence (GRADE 1C)*	

### Literature review

An integrated description and diagnosis of BDI is essential to choose the most appropriate management, which depends on the time of detection, the extent of bile duct and vascular injuries, and the underlying mechanism. These aspects must be included in the diagnostic assessment using an appropriate and specific BDI classification.

Several BDI classifications have been proposed over the years. They described different subtypes of injuries according to their severity and have taken into account the biliary tract anatomy or the level of the biliary injury; alternatively, some integrated the possible associated vascular injuries of the hepatic hilum into the description of BDI [91]. To date, there is still no consensus on a “gold standard” classification for BDIs, but there are some widely adopted classification systems, which are summarized in Table 2. Direct comparisons among the available classification systems are also difficult. Each classification has strengths and drawbacks, as they all lack the standardization of a common nomenclature.

**Table 2:** Summary of the most commonly used BDI classification systems

	BDI classification systems								
	Bismuth [92, 93]	Strasberg [90]	McMahon [94]	Bergman [95]	Csendes [97]	Stewart-Way [98, 103]	Hannover [17]	Lau [99]	ATOM [100]
**Bile leakage**									
Cystic duct leak or leaks from small ducts in liver bed		A		A	Type I		Type A	Type 1	NMBD
Occlusion of an aberrant RHD		B						Type 2	
Leak from an aberrant RHD		C							
Lateral injury to CBD < 50% diameter		D						Type 2	
Laceration > 25% of CBD			Major bile duct injury	B					
Transection of CBD or CHD			Major bile duct injury	D	Type III	Class II/III	Type D	Type 3	
Resection od more than 10 mm of the CBD					Type IV				
Tangential injury of the CBD							Type C		
Right/left hepatic duct or sectoral duct injuries								Type 4	
Laceration < 25% of CBD			Minor bile duct injury			Class I			
Laceration of cystic-CBD junction			Minor bile duct injury		Type II				
**Bile stricture**									
Stenosis of the main bile duct without injury (caused by a clip)							Type B		
CBD stump > 2 cm	Type I	EI							MBD 1
CBD stump < 2 cm	Type II	E2							MBD 2
Ceiling of the biliary confluence is intact	Type III	E3							MBD 3
Ceiling of the confluence is destroyed	Type IV	E4							MBD 4
Type I, II or III + stricture of an isolated right duct	Type V	E5							
Development of post-operative CBD stricture			Major bile duct injury	C			Type E		
**Vascular lesion**									
Right hepatic artery + RHD transected						Class IV	Type D	Type 5	VBI+

*RHD* right hepatic duct, *CBD* common bile duct, *CHD* common hepatic duct, *NMBD* non-main bile duct, *MBD* main bile duct, *VBI* vasculobiliary injury

#### How to classify BDI

Classification systems that are essentially based on the biliary injury location include the first classification published by Bismuth in 1982 [92, 93], followed by the Strasberg’s one proposed in 1995 [90], and other classification systems published by McMahon [94], Bergman [95], Neuhaus [96], and Csendes [97]. Conversely, classification systems that integrate vascular injuries into the description of BDIs are the Stewart-Way classification published in 2007 [98], the Hannover classification [17], the one proposed by Lau et al. [99], and more recently the ATOM (Anatomic, Time Of detection, Mechanism) classification published by the European Association for Endoscopic Surgery (EAES) in 2013 [100]. The ATOM integrates the Bismuth, Strasberg, Neuhaus, McMahon, Connor, and Lau classifications into a composite, all-inclusive, nominal system (Tables 2 and 3), which combines bile tract anatomical damage, vascular injury, timing of detection, and mechanism of damage in an exhaustive classification system covering all possible injuries. The EAES intended the ATOM classification as a specific effort toward standardization and transformation of BDI definitions into a unified language. Moreover, the ATOM system was thought to facilitate the collection of data for epidemiological and comparative studies, ultimately leading to a more precise determination of the true incidence of BDI incurred during LC and consequently favoring the development of preventive measures [101]. The main drawback is that it may be too complex and time-consuming to be used in routine clinical practice.

**Table 3:** ATOM classification [100]

Anatomical characteristics							Time of detection		Mechanism		
Anatomic level	Type and extent of injury					Vasculobiliary injury (yes = VBI+) and name of injured vessel (RHA, LHA, CHA, PV, MV); (no = VBI−)	Ei (de visu, bile leak, IOC)	Ep	L	Me	ED
	Occlusion		Division								
	C	P*	C	P*	LS**						
MBD											
1											
2											
3											
4											
5											
6											
NMBD											

*MBD* main biliary duct, *NMBD* non main biliary duct (Luschka duct, aberrant duct, accessory duct), *C* complete, *P*, partial, *LS*, loss of substance, *Me*, mechanical, *ED* energy driven, *VBI* vasculobiliary involvement, *RHA* right hepatic artery, LHA left hepatic artery, *CHA* common hepatic artery, *PV* portal vein, *MV* marginal vessels, *Ei* early intraoperative, *Ep* early postoperative, *L* late, *OC* intra-operative cholangiogram

Clinically, BDIs are often grouped into minor or major injuries. Minor BDIs include injuries caused by electrocautery burns or a partial cut from sharp dissection with shears and are not associated with tissue loss. These injuries can typically be repaired primarily with sutures and placement of abdominal drains in the area [102]. Conversely, major BDIs (i.e., Strasberg E) are associated with tissue loss (e.g., the common bile duct is clipped and transected) and require complex reconstruction with a Roux-en-Y hepaticojejunostomy.

#### How to describe BDI

Once BDI has occurred and been recognized during LC (approximately 25% of cases [99, 103]), a detailed and precise surgical report will be critically important to guide BDI management. The surgical report must include the indication for the surgery, the patient’s comorbidities, the operative time, the amount of blood loss, the type of injury that occurred (in detail), and the use of drainage.

The ideal report should follow the CVS schema described by Strasberg in 1995 [90]. The CVS is composed of three critical steps: (1) the visualization of the hepatocystic triangle with no exposure of the common bile duct; (2) the exposure of the lower part of the gallbladder and its separation from the liver bed; and (3) the visualization of only 2 structures that enter the gallbladder: the cystic duct and the cystic artery. The surgeon must report during which of the CVS steps difficulties were encountered and BDI occurred.

Whether a timeout during LC, prior to transecting any ductal structure, is performed should be reported. Similarly, it is important to report whether another surgeon was consulted at the time of the dissection or during the difficult steps. Any anatomical abnormality or unusual findings should be described, including:

- Bile drainage from a location other than the gallbladder
- Bile draining from a tubular structure
- A second cystic artery or large artery posterior to the cystic duct
- A short cystic duct
- A bile duct that can be traced to the duodenum
- Severe hemorrhage or inflammation.

Whenever an intraoperative biliary tract imaging technique (IOC or ICG-C [104]) is performed, these findings must also be reported, and cholangiography images should be included in the report [105, 106].

Particularly, the following should be specified:

- Failure to opacify the proximal hepatic duct or the cystic duct
- Identification of an extra bile duct, an aberrant bile duct, or duct of Luschka
- Ductal abnormalities: wide cystic duct (which may be the common bile duct), accessory bile duct, second cystic duct (which may be the common hepatic duct), and abnormal gallbladder infundibulum that may indicate that the common bile duct was dissected.

If possible, a drawing of the BDI with biliary drainage positioning (if used) could be helpful. If a videotape of the surgical procedure is available, it should be added to the report [107–109].

**Table:** 

**Q4. What are the surgical management strategies and timing for intraoperatively diagnosed BDI?**	
**Statements:**	
4.1. We recommend the selective use of adjuncts for biliary tract visualization (e.g., IOC, ICG-C) during difficult LC or whenever BDI is suspected to increase the rate of intraoperative diagnosis. The opinion of another surgeon should also be considered. *Weak recommendation, moderate quality of evidence (GRADE 2B)* 4.2. Direct repair with or without T-tube placement may be considered in cases of minor BDIs. Hepaticojejunostomy should be considered the treatment of choice in cases of major BDIs. *Strong recommendation, low quality of evidence* (*GRADE 1C)* 4.3. Early BDI repair (on-table up to 72 h) may be considered in cases of appropriate surgical indications and expertise. Referral to an HPB center should be considered if sufficient HPB expertise is not available locally. *Strong recommendation, low quality of evidence (GRADE 1C)* 4.4. Systematic immediate repair of isolated injuries of the right hepatic artery is not recommended, and the benefit/risk ratio should be evaluated carefully. *Weak recommendation, very low quality of evidence (GRADE 2C)* 4.5. The repair of complex injuries (e.g., vasculobiliary) should be delayed and not attempted intraoperatively, even by expert HPB surgeons. *Weak recommendation, low quality of evidence (GRADE 2C)*	

### Literature review

In the event of intraoperative recognition of BDI, the subsequent management is highly dependent on the injury extent and classification. The first key factor is the timing of the intraoperative recognition of BDI: the earlier the recognition, the better the outcomes [79]. Data from the nationwide GallRiks prospective registry highlighted that patients with BDI have a significantly poorer 1-year overall survival than non-injured patients (1-year mortality: 3.9% vs. 1.1%, respectively). Particularly, Cox regression analysis demonstrated that patients who had injuries with delayed detection have almost a doubled risk of mortality compared with patients who had no injury (hazard ratio, HR: 1.95; 95% confidence interval, CI: 1.12–3.37). Conversely, no difference in 1-year survival rates was observed in patients with BDIs detected perioperatively compared to those without a BDI [78]. These data demonstrate that the timing of BDI recognition matters; nevertheless, BDIs diagnosed intraoperatively represent only a limited number of cases, although the ranges reported in the literature are highly variable (25–92%) [9, 79, 102, 110, 111].

To help in the intraoperative detection and classification of BDI, several adjuncts can be used, such as intraoperative ultrasonography (IOUS), IOC, and ICG-C. Conversion to open surgery may be also considered in the event of BDI during LC [112, 113], with conversion rates that vary from 23 to 71% [102, 114]. However, conversion to open surgery is not recommended if the surgeon has sufficient experience in minimally invasive surgery to manage BDI laparoscopically.

#### Management of intraoperatively diagnosed BDI

The presence of an unexplained source of bile in the operative field must raise the suspicion of a BDI. In these cases, the use of IOC is helpful to detect BDI, although it requires additional training and longer operative times [7, 115]. A meta-analysis on 860 patients showed that the selective versus the routine use of IOC is associated with a comparable chance of detecting BDI (odds ratio, OR: 0.36; 95% CI: 0.01–8.92; z = 0.63; p = 0.53) [116]. On the contrary, its use appeared to be helpful in terms of BDI risk reduction in patients with AC (moderate or severe) [67, 78].

IOUS could be useful to evaluate vascular injuries associated with BDI and should be preferred to hilar dissection during intraoperative staging to avoid further damage [117].

ICG-C provides real-time imaging of the extrahepatic biliary tract during LC and represents a noninvasive, quick, safe, and easy-to-apply tool [64]. A recent meta-analysis of 19 studies including 772 patients explored the potential of ICG-C to identify biliary structures during LC [118]. Four studies compared the use of ICG-C to IOC in 215 patients and found no significant differences for cystic duct, common bile duct, or common hepatic duct visualization. A recent survey involving 3411 surgeons (with an average of 16.1 years of practice) highlighted how the use of adjuncts such IOC, ICG-C, or intraoperative ultrasound, either routinely or selectively during difficult cholecystectomies, is not significantly associated with a lower risk of BDIs [9]. It is important to emphasize that factors such as geographic distance between facilities, equipment, expertise, and logistics, vary significantly between institutions. Some authors proposed that in cases of suspected BDI, asking the opinion of another surgeon (physically or virtually) may be an easy, effective, and inexpensive alternative to IOC [119].

In the event of BDI detected during LC, surgeons must promptly analyze the injury and choose between an intraoperative repair or “drain now and fix later” strategy [120]. For minor BDIs (i.e., Strasberg A–D and conditionally E2), a direct repair, with or without the placement of a T-tube, and the placement of abdominal drains in the area is considered safe and appropriate [121]. This strategy is reported in 5–58% of BDI cases in the literature [102, 111, 122, 123].

If available on site, endoscopic decompression might be considered in cases of Strasberg A injury [124]. However, the recur to this strategy is blunted by the high rate of repair failure (up to 64%) [111].

For major BDIs (i.e., Strasberg E) associated with tissue loss and whenever an ischemic injury is suspected, a Roux-en-Y hepaticojejunostomy is the recommended method of reconstruction [9, 111, 122, 125–128], with the placement of a T-tube at a healthy region of the common bile duct, either proximal or distal to the injury, to decrease the incidence of future stricture formation [129]. Any dissection in the hilum may make subsequent reconstruction more difficult or cause further biliary or vascular injury. Thus, in case of insufficient experience in hepato-pancreato-biliary (HPB) surgery, it is recommended to place a drain in the right upper quadrant and transfer the patient to a center with experienced HPB surgeons [129]. Conversion to an open surgery to solely confirm diagnosis or perform injury staging is not recommended.

A recent systematic review and meta-analysis [130] considering 10 low-quality studies showed that on-table repair (by direct suture or bilioenteric anastomosis) is associated with a higher incidence, although non statistically significant, of failure than postoperative repair (60% vs. 34.1%; OR: 2.06; 95% CI: 0.89–4.73; p = 0.09). Moreover, non-expert immediate repair attempts are associated with worse outcomes than expert repair potentially compromising later revisions of the injury by a specialist [130]. As supported by another single-center cohort study on 200 patients with BDIs, on-table repair by non-HPB specialists appears to be an independent risk factor for recurrent cholangitis, biliary strictures, revision surgery, and overall morbidity [126]. On the contrary, an early referral to an HPB center can significantly decrease the rate of postoperative complications (OR: 0.24; 95% CI: 0.09–0.68; p = 0.007) and biliary strictures (OR: 0.28; 95% CI: 0.17–0.47; p < 0.001) compared to delayed referral [130]. Whenever a sufficient HPB experience is locally available, some data suggest that the earlier the repair, the better the results [79, 102, 122, 126, 131], whereas other studies support that similar and good outcomes are to be expected for on-table / early (within 72 h [128]) repair vs. postoperative repair (within 1 week [131]) when the BDI is managed by HPB surgeons or in HPB referral centers [130].

However, it must be considered that in some countries or regions, a tertiary/specialist care center may be too distant, and the “traveling surgeon” practice may be inappropriate [20, 127]. In these specific cases, it is of utmost importance to assure an optimal local management before referral, especially when, due to logistic and geographical constraints, the time prior to transport may be prolonged [20].

#### Management of concomitant vascular injuries

Because the hepatic blood supply is mainly carried by the portal vein, the interruption of the right branch of the hepatic artery alone is usually well tolerated [117, 132, 133]. Whenever an injury is recognized, the immediate repair of the right hepatic artery is not the most frequent option even in tertiary care centers, being good results only occasionally reported (i.e., no occurrence of liver infarction and uneventful follow-up) [132, 133]. Indeed, opportunities for immediate arterial repair are limited due to the low rate of injury recognition, the low number of patients affected by symptomatic liver ischemia, and the high level of technical expertise required. Moreover, an extensive imaging workup with a contrast-enhanced CT scan is mandatory prior to attempting the vascular repair. Thus, given the low clinical impact and the technical complexity of the procedure, the efficacy of arterial reconstruction remains questionable [132, 134].

Vasculobiliary injuries, defined by the presence of both biliary (bile duct obstruction or hilar plate division) and vascular injuries (hepatic artery and/or portal vein injury), lead to liver ischemia in 10% of cases [132, 133]. Their management depends on the evidence and extent of the liver injury (e.g., ischemia, necrosis, or atrophy). Their stabilization may require few weeks or months. In general, the surgical management should be delayed to allow for an accurate imaging workup and strategic planning, which involves HPB surgeons.

**Fig. 1:**
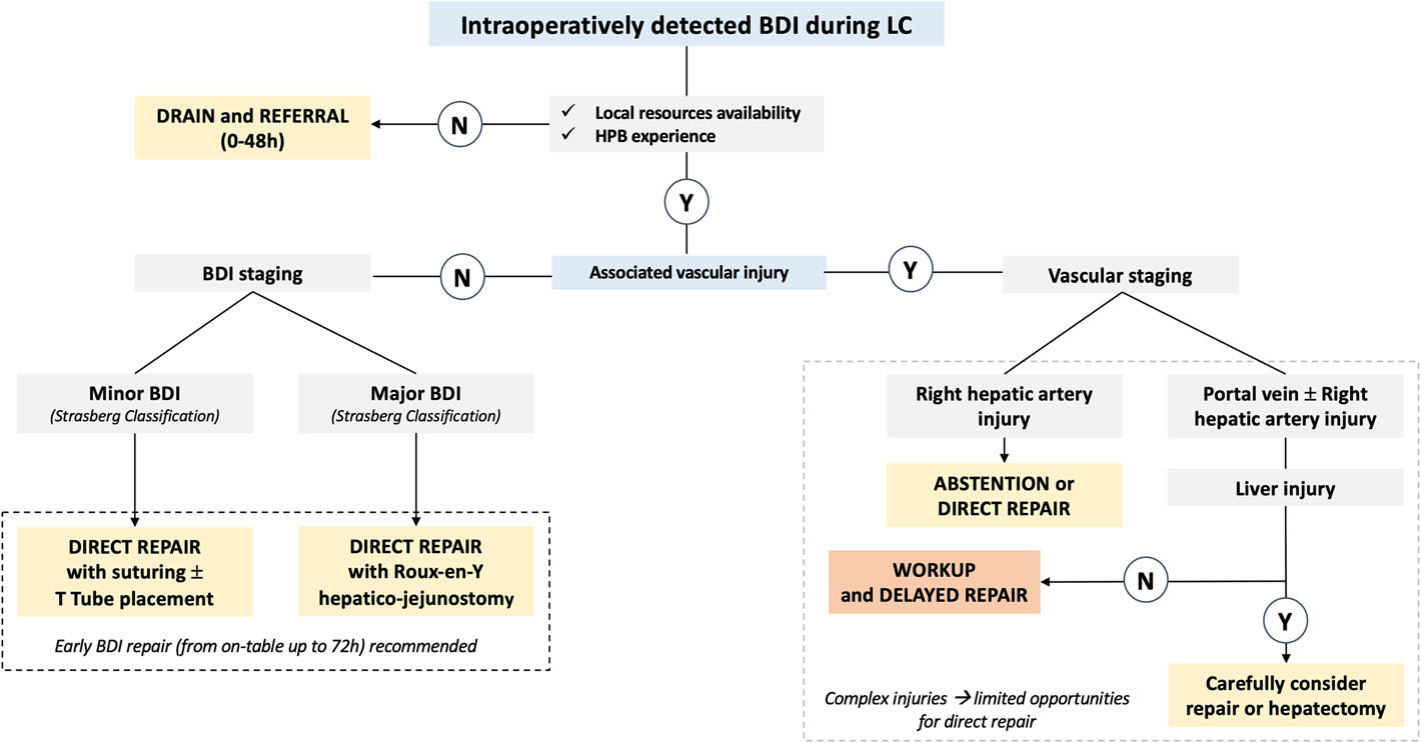
Decisional tree in case of intra-operatively detected BDI. *N stands for no*, *Y for yes*

A decision tree for the management of intraoperatively detected BDIs is displayed in Fig. 1.

**Table:** 

**Q5. What is the recommended type and duration of antibiotic regimen in cases of BDI?**	
**Statements:**	
5.1. In cases of suspected BDI during elective LC without a history of previous biliary drainage, antibiotic therapy may be considered using broad-spectrum antibiotics. *Weak recommendation, very low quality of evidence (GRADE 2C)* 5.2. In patients with previous biliary infection (i.e., cholecystitis, cholangitis) and patients with preoperative endoscopic stenting, ENBD, or PTBD at risk of developing local and systemic sepsis, broad-spectrum antibiotics (4^th^-generation cephalosporins) are recommended, with further adjustments according to antibiograms. *Strong recommendation, low quality of evidence (GRADE 1C)* 5.3. In patients with biliary fistula, biloma, or bile peritonitis, antibiotics should be started immediately (within 1 h) using piperacillin/tazobactam, imipenem/cilastatin, meropenem, ertapenem, or aztreonam associated with amikacin in cases of shock and using fluconazole in fragile patients and cases of delayed diagnosis. *Strong recommendation, low quality of evidence (GRADE 1C)* 5.4. In severe complicated intra-abdominal sepsis, open abdomen can be considered an option for patients with organ failure and gross contamination. *Weak recommendation, low and very low quality of evidence (GRADE 2C)*

### Literature review

To our knowledge, no study has specifically investigated the indications, duration, and type of antibiotic therapy in cases of BDI. In the absence of specific scientific data, the following recommendations are adapted from published literature and guidelines about the management of biliary infections and abdominal sepsis [135–142].

In general, depending on the timing of discovery and presentation of BDI, consistent literature supports the initiation of antibiotic therapy as a complement to source control strategies in early or late identification of BDIs. However, no consensus exists on the duration of antibiotic treatment before or after gallbladder surgery [143].

#### Antibiotic therapy in case of intraoperatively diagnosed BDI

In patients with previous biliary infections (e.g., cholecystitis, cholangitis) and patients with preoperative instrumentation such as endoscopic stenting at ERCP/sphincterotomy, endoscopic nasobiliary drainage (ENBD), or percutaneous transhepatic biliary drainage/cholangiography (PTBD/PTC), there may be preexisting bactobilia. Consequently, bile flow into the peritoneal cavity may lead to local or systemic sepsis. Thus, intraoperative antibiotic coverage must be initiated or continued in case an antibiotic prophylaxis has been already administered. Bile culture is mandatory to narrow the coverage spectrum and prevent antibiotic resistance. Treatment should last no more than 24h [136, 137]. The recommended antibiotics include cefazolin, cefamandole, or cefuroxime (to be substituted by gentamicin and clindamycin in case of allergy) [136, 137]. In case of infection and ongoing drainage, the following antibiotics can be considered: piperacillin/tazobactam, ceftriaxone, or other 4^th^-generation cephalosporins [144], for a minimum of 5 days of treatment.

#### Antibiotic therapy in case of postcholecystectomy biliary ductal stenosis

In the case of biliary obstruction without bile leak or signs of sepsis, antibiotic therapy may not be required. However, the majority of patients with biliary obstruction have infected bile and grow bacteria from cultures even when clinical cholangitis is not yet present. Sepsis may occur after biliary instrumentation and drainage using endoscopic stenting, ENBD, or PTBD. Antibiotic prophylaxis is appropriate and recommended to prevent the occurrence of healthcare-associated acute cholangitis, especially in the setting of predictable incomplete drainage [145].

#### Antibiotic therapy in case of biliary leakage

The first priority in case of bile leakage is “source control” and early “goal-directed therapy” [140]. Antibiotic therapy should be initiated as soon as evidence of cholangitis or infected fluid collections appears [146]. In patients without shock, radiological and bacteriological sampling can be performed to obtain definitive diagnostic studies before starting parenteral antibiotic therapy. A 6-h delay period might be tolerated. In the presence of severe sepsis or shock, the investigation window should be substantially shortened, and broad-spectrum antibiotics should be started within 1 h of the initiation of signs and symptoms. Treatment should be adapted according to bile culture findings [136, 137]. In the worst cases of severe complicated intra-abdominal sepsis, open abdomen (OA) therapy for optimal source control may be considered [147, 148], although the biological basis for OA in such cases is currently being subjected to rigorous scientific scrutiny [149].

In the case of external biliary fistula without intraperitoneal collection, antimicrobial therapy might not be necessary if infectious signs are absent. The natural history of an external fistula depends on the anatomical subtype of injuries. In complex BDIs requiring delayed surgical repair, complete healing of the fistula is an absolute prerequisite for surgery. During the waiting period, several patients may experience cholangitis. The Tokyo guidelines published in 2018 for the severity grading and management of cholangitis may be applicable [144]. Biliary drainage, most often using PTBD, should be placed in cases of uncontrolled or recurrent cholangitis. Parenteral broad-spectrum antibiotics should be started and subsequently adapted to bile and blood cultures [150, 151]. Management of biloma and peritonitis requires percutaneous drainage and surgery, respectively. In the case of cholangiolytic abscesses, which are usually small and multiple, parenteral antibiotics and biliary drainage (endoscopic or percutaneous) may be indicated. A large cholangiolytic abscess not responding to parenteral antibiotics within 48–72 h may require imaging and US- or CT-guided percutaneous needle aspiration or catheter drainage. The antibiotics most often used in cases of biliary peritonitis are piperacillin/tazobactam, imipenem/cilastatin, meropenem, ertapenem, or aztreonam associated with amikacin in cases of associated shock and fluconazole in cases of fragility or delayed diagnosis.

The optimum duration of antibiotic therapy in the setting of biliary infection is a matter of debate. According to the Tokyo Guidelines [144], an additional 4 days of antibiotic therapy is required after source control of cholangitis by decompression of the biliary tree. Treatment should be continued for 2 weeks in the presence of *Enterococcus* or *Streptococcus* to prevent the risk of infectious endocarditis. Frailty and comorbid factors must also be accounted for in the titration of therapy. However, other studies showed that only 3 additional days are sufficient to reduce the risk of recurrence [152, 153]. For biloma and generalized peritonitis, a treatment of 5–7 days should be considered [140].

**Table:** 

**Q6. Which are the clinical, biochemical, and imaging investigations required for the postoperative diagnosis of BDI?**	
**Statements:**	
6.1. We recommend a prompt investigation of patients who do not rapidly recover after LC, with alarm symptoms being fever, abdominal pain, distention, jaundice, nausea, and vomiting (depending on the type of BDI). *Weak recommendation, low quality of evidence (GRADE 2C)* 6.2. The assessment of liver function tests, including serum levels of direct and indirect bilirubin, AST, ALT, ALP, GGT, and albumin, is suggested in patients with clinical signs and symptoms suggestive of BDI after LC. In critically ill patients, the serum levels of CRP, PCT, and lactate may help in the evaluation of the severity of acute inflammation and sepsis and in monitoring the response to treatment. *Weak recommendation, low quality of evidence (GRADE 2C)* 6.3. Abdominal triphasic CT is suggested as the first-line diagnostic imaging investigation to detect intra-abdominal fluid collections and ductal dilation. It may be complemented with the addition of CE-MRCP to obtain the exact visualization, localization, and classification of BDI, which is essential for planning a tailored treatment. *Weak recommendation, moderate quality of evidence (GRADE 2B)*	

### Literature review

BDIs should be suspected and diagnosed as early as possible in patients who do not promptly recover after LC. The postoperative diagnosis of BDI is based on the evaluation of signs and symptoms, laboratory tests, and imaging studies.

#### Clinical signs and symptoms of BDI

The most frequent complaints of patients with BDI are persistent abdominal pain, abdominal distension, nausea and/or vomiting, fever, and jaundice [18]. The BDI clinical presentations are related to the type of injury. The two most frequent clinical scenarios are bile leakage and bile duct obstruction [154]. In patients with a bile leak, an early visible sign is the presence of bile from the drain or surgical incision. If the subhepatic region is not drained, a perihepatic bile collection (biloma), abscess, or biliary peritonitis may develop with corresponding clinical signs. Generally, jaundice is not observed or is mild in these cases because cholestasis does not occur [18, 154–156]. In patients with biliary strictures, symptoms are often delayed. Cholestatic jaundice with choluria, fecal acholia, and pruritus are the most common clinical signs and symptoms. If cholangitis develops, fever with chills is typically associated with jaundice [154–157]. Recurrent cholangitis is the main consequence of bile duct stricture, hepatic injury and dysfunction from complete bile duct occlusion. Sepsis and multiorgan failure may develop in both clinical settings.

When BDI is not identified intraoperatively or during the first postoperative week, patients may have an insidious evolution with relapsing abdominal pain, cholangitis, and bile collections. A late diagnosis, which sometimes is made years after surgery following multiple ineffective attempted repairs or inappropriate management, may result in increased complexity of bile duct repair. Moreover, even if successfully managed, the patient’s quality of life and survival may be impaired [158]. Indeed, the clinical course of undiagnosed or unrepaired BDI can evolve to secondary biliary cirrhosis with portal hypertension, liver failure, and, ultimately, death [18].

#### Biochemical tests for the diagnosis of BDI

After elective LC, laboratory tests are not routinely required because mild to moderate elevations in hepatocellular enzymes are frequently observed during the postoperative period but have no pathological meaning; CO_2_ pneumoperitoneum seems to be the main reason for these changes [159, 160].

In clinical practice, surgeons should consider postoperative biochemical investigations whenever difficulties were encountered during the intervention or in the presence of postoperative clinical signs suggestive of complications. These are performed to aid in the diagnosis [154, 157, 161]. Ben-Ishay et al. [161] evaluated the utility of post-LC blood examinations by retrospectively analyzing the chart data of approximately 340 patients undergoing LC and confirmed that they may be useful to make diagnoses and lead to early interventions in complicated cases. Blood tests were most often obtained in elderly patients and those who had prolonged surgery, multiple drains, and longer hospital stays.

Serum levels of direct and indirect bilirubin, aspartate aminotransferase (AST), alanine aminotransferase (ALT), alkaline phosphatase (ALP), gamma-glutamyl transpeptidase (GGT), and albumin, as well as a complete blood count (CBC), are usually measured to diagnose iatrogenic BDI [154, 157]. In BDI patients, liver function tests and cholestatic enzymes may either be elevated, supporting the clinical suspicion, or remain within the normal ranges. In the case of stenosis or complete occlusion of the bile duct, bilirubin values increase, whereas no elevation or only a slight elevation may be observed as a result of peritoneal bile absorption in the presence of bile leakage [99]. In the very early stages, cholestasis markers are increased, but there is no significant hepatic damage; therefore, aminotransferases are not increased. Early in the initial postoperative course, the determination of ALP and total bilirubin is not sensitive [162].

Biomarkers, such as C-reactive protein (CRP), procalcitonin (PCT), and serum lactate, can help to evaluate the severity of the inflammation or sepsis and provide a baseline to follow the therapeutic response [163, 164]. PCT, CRP, and lactate levels can also be used to predict fatal progression in septic patients and are associated with poor outcomes and increased mortality [163, 164].

#### Imaging for postoperative diagnosis of BDI

The role of imaging is to establish the BDI diagnosis, delineate the type and extent of the injury, and plan the appropriate intervention.

Ultrasonography (US) represents the primary noninvasive and easily available diagnostic tool that allows for the detection of intra-abdominal fluid collections, dilation of the biliary ducts, and possibly associated vascular lesions by using Doppler evaluation [154, 157, 165]. Abdominal triphasic CT scanning is useful to identify the possible presence of focal intra- or perihepatic fluid collections, ascites, biliary obstruction with upstream dilation, and long-term sequelae of a long-standing bile stricture, such as lobar hepatic atrophy or signs of secondary biliary cirrhosis. CT can also identify associated vascular lesions, such as injury to the right hepatic artery [154, 157]. The sensitivity of CT is superior to that of US, especially for the detection of small fluid collections and associated vascular complications [166–168]. US provides good anatomic and contrast resolution, but CT has higher spatial resolution and better identification of fluid collection morphology and site; CT is also essential to define collections that require percutaneous or surgical drainage. However, neither US nor CT examinations can reliably distinguish bile leaks from other postoperative fluid collections, such as blood, pus, or serous fluid, because of their similar densities. Neither can establish the precise location or the active state of a bile leak because the bile collection site may not be separate from the leak site and occasionally may even be intrahepatic [169].

Hepatobiliary scintigraphy (HS) has two potential advantages over US and CT. It seems to be more sensitive and specific than US or CT in detecting bile leaks [170], and in addition to confirming the presence of a bile leak, it can identify the relationship between the leak and any fluid collection as well as show the primary route of bile flow [171]. Despite this, it is frequently necessary to complete HS with additional investigations. In fact, HS can provide functional information demonstrating the presence of an active leak, but its spatial resolution is poor, and the identification of the leak site can be challenging [169, 172]. Other pitfalls of HS are that extrabiliary structures are not visualized, so no information about them can be obtained, it has poor sensitivity in patients with hepatic dysfunction and large bile duct defects with preferential bile flow in a path of least resistance, and it may not show activity in the duodenum and thus a bile leak may be misinterpreted as a complete bile duct obstruction [169].

The use of ERCP and PTC can identify a continuing bile leak, provide exact anatomical diagnosis, and allow, at the same time, the treatment of the injury by appropriately decompressing or dilating the biliary tree. ERCP can be applied to treat bile leaks using internal stents. Success using this technique may be more likely if the injury to the duct is < 5 mm, if the injury is extrahepatic, and when there is no associated abscess or biloma [173]. In the case of ERCP failure, PTC is a valuable option to accurately depict the location and nature of BDI and to perform an extraluminal percutaneous endoscopic rendezvous procedure with stent placement to restore continuity of the bile duct [174–176].

On the other hand, ERCP and PTC are invasive techniques that are associated with a nonnegligible risk of complications, including severe acute pancreatitis (mainly after ERCP), bleeding, and cholangitis (after PTC) [177, 178]. Other disadvantages are the lack of detection of extrabiliary abnormalities and the non-visualization of ducts upstream or downstream from an obstructing lesion (e.g., stricture, stone). Moreover, PTC can be technically difficult because intrahepatic bile ducts are usually not dilated [170].

Magnetic resonance cholangiopancreatography (MRCP) represents the “gold standard” for a complete morphological evaluation of the biliary tree, as it is noninvasive, does not use ionizing radiation, and provides excellent anatomical information regarding the biliary tree anatomy proximal and distal to the level of injury [154, 157, 169]. MRCP combined with dynamic contrast-enhanced magnetic resonance using a hepatocyte-selective contrast agent with biliary excretion allows for the functional assessment of the biliary tree, and thus, the detection and localization of bile leaks with an accuracy close to 100% [179]. In the past, the use of mangafodipir trisodium as a contrast agent primarily excreted via bile — now withdrawn from the EU Market — was shown to be useful for both diagnosing a bile leak and identifying the source of the leak by direct visualization of contrast material extravasation into fluid collections [179, 180].

Several authors [181–185] confirmed that the additional use of contrast-enhanced MRCP (CE-MRCP) using 3D and 2D T1-weighted images acquired at the hepatobiliary phase after hepato-specific contrast agent injection improves the accuracy of bile anatomy depiction and bile leak detection. In a series of 99 patients — including 24 followed after cholecystectomy, 20 after surgical reconstruction of traumatic BDI, and 16 after hydatid cystectomy — the use of CE-MRCP increased the sensitivity, specificity, and accuracy, with respective ranges (depending on the bile leak etiology) of 76–82%, 100%, and 75–91% compared to 53–63%, 51–66%, and 55–63% observed with conventional MRCP [183]. The optimal timing for hepatobiliary phase acquisitions with CE-MRCP appears to range between 60 and 90 min when looking for bile leaks [182, 186].

In the post-liver transplant setting, Boraschi et al. [187] studied 384 MRCP examinations in 232 patients. The reported sensitivity, specificity, positive predictive value, and negative predictive value for the detection of BDI were 99%, 96%, 99%, and 97%, respectively. One considerable MRCP limitation is the poor opacification of bile ducts in the presence of obstruction and unreliable depiction of the more peripheral intrahepatic bile ducts [179].

**Table:** 

**Q7. What are the surgical management strategies and timing for postoperatively diagnosed BDI?**	
**Statements:**	
7.1. In the case of minor BDIs (e.g., Strasberg A–D), if a drain is placed after surgery and a bile leak is noted, an observation period and non-operative management during the first hours is an option. If no drain is placed during surgery, percutaneous treatment of the collection with drain placement can be useful. *Weak recommendation, low quality of the evidence (GRADE 2C)* 7.2. For minor BDIs, if no improvements or worsening of symptoms occurs during the clinical observation period after percutaneous drain placement, endoscopic management (by ERCP with biliary sphincterotomy and stent placement) becomes mandatory. *Strong recommendation, low quality of the evidence (GRADE 1C)* 7.3. In major BDIs (e.g., Strasberg E1–E2) diagnosed in the immediate postoperative period (within 72 h), we recommend referral to a center with expertise in HPB procedures if that expertise is locally unavailable. An urgent surgical repair with bilioenteric anastomosis Roux-en-Y hepaticojejunostomy could then be performed. *Strong recommendation, low quality of the evidence (GRADE 1C)* 7.4. In major BDIs diagnosed between 72 h and 3 weeks, we recommend percutaneous drainage of the fluid collections whenever present, targeted antibiotics, and nutritional support. During this period, an ERCP (sphincterotomy with or without stent) can be considered to reduce the pressure gradient in the biliary tree, and a PTBD could be useful for septic patients with a complete obstruction of the common bile duct. After a minimum of 3 weeks, if the patient’s general conditions allow and the acute or subacute situation is resolved (e.g., closure of the biliary fistula), Roux-en-Y hepaticojejunostomy should be performed. *Weak recommendation, low quality of the evidence (GRADE 2C)* 7.5. When major BDIs are recognized late after the index LC and there are clinical manifestations of stricture, Roux-en-Y hepaticojejunostomy should be performed. *Weak recommendation, low quality of the evidence (GRADE 2C)* 7.6. When major BDIs present as diffuse biliary peritonitis, urgent abdominal cavity lavage and drainage are required as the first step of treatment to achieve infection source control. *Strong recommendation, low quality of the evidence (GRADE 1C)*	

### Literature review

**Fig. 2:**
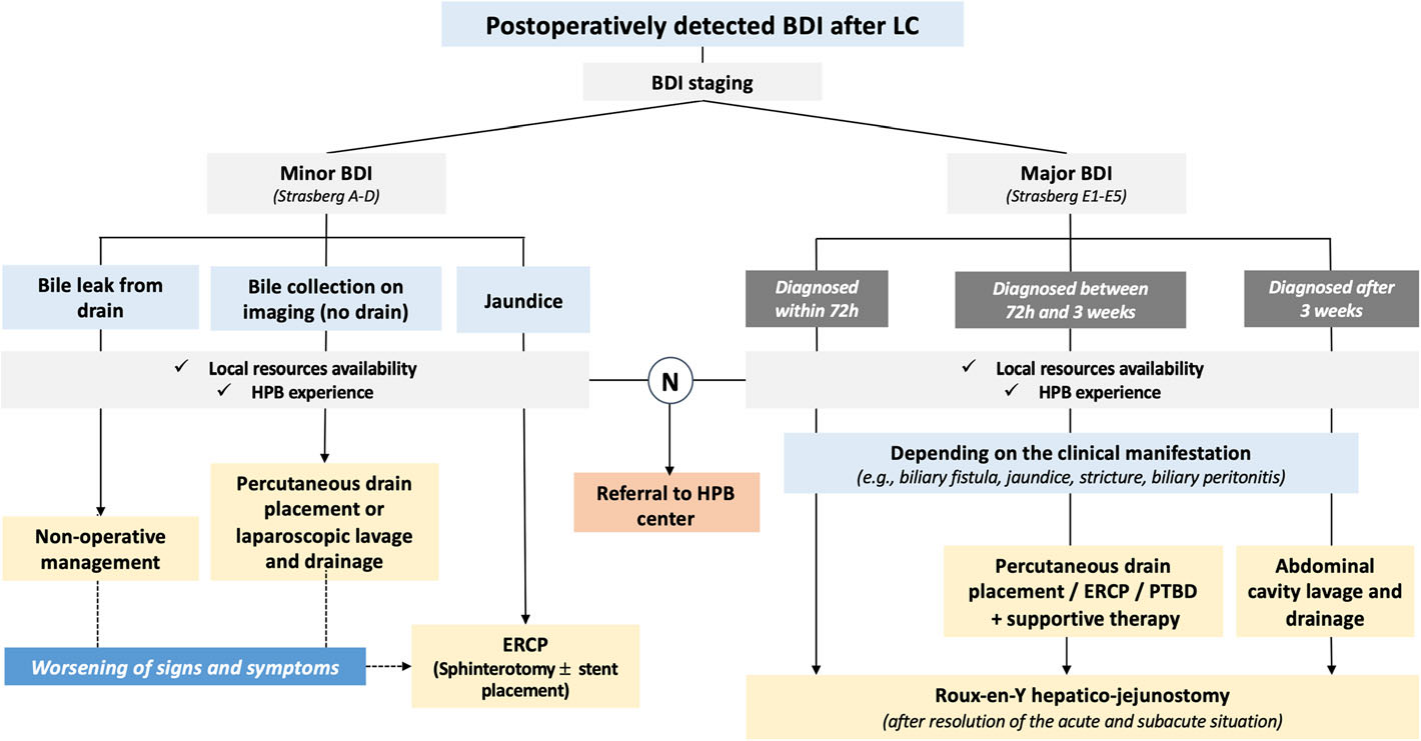
Decisional tree in case of post-operatively detected BDI. *N stands for no*

With the great majority of BDIs being detected and diagnosed postoperatively [188, 189], the type of management must be chosen based on multiple criteria, including the complexity of the biliary injury, the severity of clinical presentation, the patient’s fitness and comorbidities, and the availability of a skilled surgeon with expertise in HPB surgery. In all cases, a multidisciplinary approach involving interventional radiologists, gastroenterologists, and surgeons is advocated [19, 190]. Figure 2 depicts a decision flowchart for cases of postoperatively detected BDI.

#### Management of minor BDIs

Minor BDIs (e.g., Strasberg A-D [90, 92]) require a step-up approach once diagnosed. Common symptoms (e.g., abdominal pain or distension, fever, nausea), when noted in the postoperative period, may herald postoperative complications. In the presence of bile leakage from the drain, observation and non-operative management are advisable during the first hours [191]. If no drain was placed after surgery and imaging reveals a bile collection with suspicion of minor BDI (such as a cystic duct leak or duct of Luschka), percutaneous drainage of the collection may be the definitive treatment [126]. Several case series have reported the feasibility of drainage under endoscopic ultrasound guidance [192], but more data are needed before this approach may be recommended in this specific clinical situation.

If no improvements or worsening of symptoms occur, endoscopic management becomes mandatory [126, 193]. The same is true for low output biliary fistulas (i.e., a bile leak from the liver bed such as a Luschka’s duct) [193–195]. Various endoscopic treatments (i.e. biliary stenting, endoscopic biliary sphincterotomy, and nasobiliary drainage) are highly effective to treat biliary leaks, except in the case of transection of the common bile duct or common hepatic duct. The time lapse between biliary injury and endoscopic treatment does not seem to significantly impact on the treatment outcomes [196].

#### Role of ERCP in BDI management

ERCP is the key tool in BDIs management because it allows the identification of the site of bile leak and, most importantly, allows internal biliary drainage if the diagnosis of minor BDI is confirmed. Moreover, incidental diagnoses, such as choledocholithiasis or bile duct stricture, may also be treated in a single procedure. For this reason, ERCP is nowadays widely recommended as first-line therapy for postoperative biliary leaks [197].

The reported success rate of ERCP in this situation ranges between 87.1% and 100%, depending on the grade and the location of the leak [198–204]. Bile leaks are divided into categories: (1) low grade, where the leak can only be identified after complete opacification of the intrahepatic biliary system; and (2) high grade, where the leak can be observed before intrahepatic opacification [203]. Leaks that respond more favorably to endoscopic treatment are those located at the end of a cystic duct stump or from a duct of Luschka, usually associated with low output [197].

The limits of the endoscopic diagnosis concern the lack of visualization of aberrant or sectioned bile ducts (i.e., an aberrant right hepatic biliary duct) and the difficulty in visualization of intra-hepatic proximal leaks. Endoscopic management should be preferred when there is at least partially documented continuity of the BDI (at the MRCP) or a very close proximity of the two biliary stumps (the proximal and the distal stumps); these are the conditions in which attempting endoscopic repair with the multistenting strategy [205].

The main goal of endoscopic therapy is to reduce the transpapillary pressure gradient to facilitate preferential bile flow through the papilla as opposed to the site of the leak, providing time to the biliary tree injury to heal. This is most commonly achieved by placing a transpapillary stent. Temporary naso-biliary drainage showed a similar efficacy when compared to plastic stents but has a lower patient compliance, so it should not be considered as the first choice [206]. There is little consensus on the role of sphincterotomy alone in the management of these patients [207, 208]. Avoiding sphincterotomy may minimize the risk for immediate (e.g., bleeding or perforation) and long-term complications (e.g., cholangitis or pancreatitis) [209]. The most frequent approach is the combination of biliary sphincterotomy with the placement of plastic stents or fully/partially covered metal stents, which is associated with a high success rate in low-grade biliary leaks [126, 199, 202–204, 210, 211], and it is deemed even more effective in cases of high-grade leaks [199–201, 203]. Although less investigated in the literature, long-term (at 10 years) outcomes of endoscopic treatment with stent placement appeared to be good and effective in patients with postoperative biliary strictures [212–214].

Plastic stents are recommended to be placed to treat bile duct leaks [201]. For refractory bile leaks, fully covered self-expanding metal stents were demonstrated to be superior to multiple plastic stents in a non-randomized trial [215]. Stents are left in place for approximately 4 to 8 weeks in many studies and removed if retrograde cholangiography shows the resolution of the leakage.

The first-line approach to benign biliary strictures complicating cholecystectomy is endoscopic, as well. When recognized early in the post-operative period, strictures are often due to surgical trauma (e.g., energy device) and associated with bile leak. These strictures respond to endoscopic treatment more favorably than fibrotic strictures, which have a delayed diagnosis. Temporary placement of multiple plastic stents over a long period of time is the preferred treatment, with a success rate ranging from 74 to 90%, but with a recurrence rate as high as 30% within 2 years from stent removal [212, 216, 217]. In case of post-cholecystectomy bile strictures located > 2 cm from the main hepatic confluence, fully covered SEMS can be an alternative to plastic stents [201].

When ERCP is unsuccessful or not feasible, PTBD becomes an alternative. Moreover, PTBD can be useful for septic patients with a complete obstruction of the common bile duct as part of the multidisciplinary approach when ERCP fails or when surgical repair failures need to be treated (i.e., stricture of the hepaticojejunostomy). PTBD in the presence of bile leakage may be more difficult as a result of non-dilated bile ducts but still leads to a technical success of 90% and a short-term clinical success of 70–80% in expertise centers [214, 218, 219].

#### Management of major BDIs

In the case of major BDIs (e.g., Strasberg E1–E5) in which there is a complete loss of common and/or hepatic bile duct continuity, carefully planned surgical treatment is required. Even when an endoscopic approach has been performed, high-grade bile leaks are difficult to manage successfully [191] and represent an independent risk factor for morbidity [199]. Early aggressive surgical repair (performed within 48 h from diagnosis) seems to guarantee good results, avoid the onset of sepsis, and provide advantages in terms of reduced costs and rate of hospital readmissions [195, 220, 221]. On the other hand, after 48–72 h, while inflammation tends to decrease, the phase of proliferation and healing begins and further complicates surgical repair. A key point is the technical contribution of the surgeon [126, 194, 222]: several studies have emphasized higher rates of postoperative failure, morbidity, and mortality when a primary surgeon without HPB expertise attempts to repair the injury [53, 189, 223–225]. Accordingly, in the case of a lack of HPB experience, referral to a tertiary care center immediately after diagnosis is essential to ensure early surgical repair with Roux-en-Y hepaticojejunostomy [90], which showed superior outcomes at 5 years compared to late repairs [126]. An end-to-end anastomosis may be performed if the loss of continuity makes it technically possible, but this approach is associated with increased failure rates [195, 226]. Regardless of the technique used, tension-free bilioenteric anastomosis with good mucosal apposition and vascularized ducts is the mainstay of treatment [227]. Recently, robotic procedures have been suggested due to enhanced visualization, better tissue handling, and more precise surgery [228]. In the presence of increased tissue fragility, the expertise of the HPB surgeon in the tertiary care center is likely to improve the final results and consequently the long-term outcomes [194, 195, 229].

When BDIs are recognized during the early postoperative period (within 2 weeks), persistent tissue injury from ongoing inflammation and the absence of bile duct dilatation appear to negatively influence the results and often lead to late strictures [229]. When the diagnosis is not immediate or logistic constraints limit referral to a tertiary care center, the “drain now, fix later” algorithm [120] with percutaneous drainage of the biloma [193], targeted antibiotic therapy, and nutritional support seems to be the best approach. After clinical stabilization, delayed surgical treatment can be safely performed [20]. When a major leak results in progressive biliary peritonitis, urgent surgical intervention is required with laparoscopic lavage of the abdominal cavity and drain placement [229]. Once the patient has been successfully stabilized, several weeks (2–3 weeks) are usually required for the resolution of the acute inflammatory phase. Accordingly, this time will allow for lowering the risks associated with extensive reconstructive surgery by reducing inflammation and guaranteeing the assessment of the extent of ischemic injury resulting from associated vascular injuries (right hepatic artery lesions have been recognized in 25% of BDIs) [111, 126, 133, 222, 229, 230]. Similarly, a 1-week delay in the diagnosis of major BDIs suggests the need for a “timeout” of 2–3 months before intervention [105]. Combined BDI and hepatic artery injuries further increase the morbidity and mortality of BDIs and may necessitate an early referral to a specialized HPB center. Combined repair may avoid ischemic damage to the liver parenchyma and the risk of leakage or stricture of the bilioenteric anastomosis. When an injury is not promptly recognized, hepatic necrosis, atrophy, or even abscess within the ischemic parenchyma may occur, which would ultimately require liver resection with bilioenteric anastomosis [117, 231, 232]. Roux-en-Y bilioenteric anastomosis represents the gold standard treatment for major BDIs and is ideally performed during the immediate postoperative period (within 72 h). However, late repair should be considered after the resolution of acute or subacute situations and the closure of a biliary fistula on dilated bile ducts. Indeed, in these complicated clinical scenarios, mortality and morbidity rates after late repair are significantly lower than rates after immediate and early repair: 0.8% vs. 2.8% vs. 2.2% and 14.3% vs. 39.2% vs. 28.7%, respectively [111]. Furthermore, the need for a second procedure, that is, failure of the first repair, appeared to be higher after immediate and early repair than after late repair: 56.7% vs. 40.7% vs. 6.8%, respectively [111, 233]. However, the E-AHPBA multicenter study showed, after multivariate regression analyses, that the timing of biliary reconstruction with hepaticojejunostomy does not have any impact on severe postoperative complications, the need for reintervention, or liver-related mortality, leaving advisable the choice of an individualized treatment strategy after iatrogenic BDI [234].

## Outcomes of BDI treatment

As described above, BDI diagnosis can be made early or late during the postoperative period. We reviewed the literature concerning the management of BDIs suspected and diagnosed postoperatively, leaving aside the clinical scenarios in which BDIs are diagnosed or suspected years after the index surgery due to the presence of biliary sequelae of unrecognized injuries [235]. These scenarios are beyond the scope of the present WSES recommendations, but it is important to emphasize that the evaluation of BDI management and repair outcomes should be pursued in the long term, since late complications, such as post-cholecystectomy biliary strictures, recurrent cholangitis, and secondary biliary cirrhosis, may occur [236, 237].

Although the literature is mainly based on case series, the treatment of BDIs is generally considered as successful [214, 238–243]. In a series of 31 patients with BDI (of which 83% were major BDIs) referred to be treated in a tertiary care institution, surgical intervention (i.e., duct-to-duct anastomosis or biliary-enteric reconstruction) represented the most frequent treatment followed by naso-biliary drainage, drainage-observation, and endoscopic sphincterotomy with biliary stenting. The overall success rate was 83.3% in the early period; however, 10 patients (32.3%) had late postoperative complications (stricture and cholangitis), and of these, 3 required endoscopic stent placement, and 7 patients underwent a biliary diversion with Roux-en-Y hepaticojejunostomy. Only one out of 24 patients with long-term follow-up developed biliary cirrhosis, supporting satisfactory long-term outcomes of BDI treatments [244].

Studies specifically investigating the long-term outcomes of Roux-en-Y hepaticojejunostomy with biliary-enteric anastomosis performed for major BDIs also support overall good outcomes [214, 239]. However, relevant postoperative complication rates are reported. Based on a recent review of the literature, the incidences of anastomotic strictures vary between 4.1% and 69%, with most studies reporting an incidence of 10–20%, and a median time to stricture formation of 11-30 months. Associated vascular injury, level of BDI, sepsis or peritonitis, and postoperative bile leakage have been shown to be associated with worse outcomes [214]. The reported incidences of biliary cirrhosis after BDI treatment varies between 2.4% and 10.9 %[214]. Mortality rate after BDI is also considerable: BDI-related mortality varies between 1.8% and 4.6%, with some evidence supporting an increased mortality of 8.8% in BDI patients compared to the expected age-adjusted death rate after 20 years [245]. Finally, most studies suggest that BDIs have a detrimental impact on health-related quality of life when compared to patients undergoing uneventful cholecystectomy. Impaired quality of life, particularly in terms of work-related limitations, loss of productivity, and increased use of disability benefits, has been reported even years after BDI treatment [214]. Thus, the best management strategy will be identified as the one associated with the more stable and predictable results over the follow-up.

## Medicolegal aspects

In Europe, approximately 19-32% of patients with BDIs are involved in a litigation claim, which translates into medical liability of the operating surgeon and/or significant payouts [214, 246]. These may represent a further argument to support the management of most BDIs in tertiary expert centers. Moreover, these data highlight the importance of peroperative informed consent, in which the possibility of severe complications, like BDI, is adequately explained to the patient. Straightforward and honest communication once the BDI has occurred and detected is also crucial.

In this perspective, we suggest implementing a “synoptic surgery reporting,” which represents a standardized reporting of data fundamental to tracking variations in care, evaluating the quality of care, and finally, improving patient outcomes and cost-effective treatments [247].

## Conclusions

LC is one of the most common operations a general surgeon performs in elective and emergency settings worldwide. BDI during LC is a severe complication that requires prompt diagnosis and specific treatment to avoid further morbidity and mortality. Practice guidelines have been proposed to prevent BDIs during LC [5], whereas BDI detection, classification, and management, once they occur, remain basically unstandardized. It is critical to have a plan if an injury is detected intraoperatively and to follow a standardized protocol in case of delayed diagnosis during the postoperative period. The present guidelines offer a thorough overview of the current literature about the key aspects of BDI detection and treatment strategies in various clinical situations. They are the results of international and multidisciplinary work promoted by the World Society of Emergency Surgery culminating in a consensus conference where all proposed statements and recommendations were approved by the worldwide contributing experts (a summary of all statements and recommendations is provided in Table 4). We must acknowledge that, despite the large number of publications on the topic, evidence was often derived from retrospective, moderate- to low-quality studies. However, the broad consensus reached by the expert panel allowed proposing recommendations in most cases.

**Table 4:** Summary of the consensus statements on BDI detection and management

Topic	Statements	Grade
**Minimize the risk of BDI during LC**	1.1. The use of the CVS during LC (achieving all 3 components) is the recommended approach to minimize the risk of BDIs.	1C
	1.2. If the CVS is not achievable during a difficult LC, a bailout procedure, such as STC, should be considered.	1B
	1.3. Conversion to open surgery may be considered during a difficult LC whenever the operating surgeon cannot manage the procedure laparoscopically. However, there is insufficient evidence to support conversion to open surgery as a strategy to avoid or reduce the risk of BDI in difficult LCs.	2B
	1.4. Intraoperative IOC is useful to recognize bile duct anatomy and choledocholithiasis in cases of intraoperative suspicion of BDI, misunderstanding of biliary anatomy, or inability to see the CVS, but routine use to reduce the BDI rate is not yet recommended.	2A
	1.5. Intraoperative ICG-C is a promising noninvasive tool to recognize bile duct anatomy and vascular structures, but routine use to reduce the BDI rate is not yet recommended.	2C
	1.6. In patients presenting with AC, the optimal timing for LC is within 48 h, and no more than 10 days from symptom appearance.	1A
	1.7. In patients with at-risk conditions (e.g., scleroatrophic cholecystitis, Mirizzi syndrome), an exhaustive preoperative work-up prior cholecystectomy is mandatory in order to discuss and balance the risks/benefits ratio of the procedure.	2C
**BDI rates and review of current practice in general surgery unit**	2.1. Based on large nationwide databases and systematic reviews of the literature, major BDIs occur in 0.1% of elective LC and 0.3% of emergency LC. If considering all types of BDIs, rates are 0.4% and 0.8% for elective and emergency settings, respectively. When a surgical team experiences an increased rate of BDIs, a careful review of the current practice is mandatory to critically analyze the possible causes and implement educational, training, and technical solutions to improve the standards of care.	1C
**BDI classifications** **BDI reporting**	3.1. We recommend knowing Strasberg’s classification, which remains the most commonly used classification for BDIs, and the ATOM classification, which represents the most recent and complete classification; the implementation of the ATOM classification should be promoted in the near future.	1C
	3.2. The ideal operative report must maximize the amount of intraoperative detail given to describe the BDI. The following should minimally be included: - The clinical context and indication for cholecystectomy - Intraoperative findings - The anatomical landmarks of the CVS - Any anatomical variation of the biliary tract - Cholangiography findings (if performed) - Operative data (e.g., operative time, blood loss, energy device used, need for conversion) - Drawing of the BDI with biliary drain placement (if used) - Videotape of the procedure (whenever available).	1C
**Intraoperatively detected BDI management**	4.1. We recommend the selective use of adjuncts for biliary tract visualization (e.g., IOC, ICG-C) during difficult LC or whenever BDI is suspected to increase the rate of intraoperative diagnosis. The opinion of another surgeon should also be considered.	2B
	4.2. Direct repair with or without T-tube placement may be considered in cases of minor BDIs. Hepaticojejunostomy should be considered as the treatment of choice in cases of major BDIs.	1C
	4.3. Early BDI repair (on-table up to 72 h) may be considered in cases of appropriate surgical indications and expertise. Referral to an HPB center should be considered if sufficient HPB expertise is not available locally.	1C
	4.4. Systematic immediate repair of isolated injuries of the right hepatic artery is not recommended, and the benefit/risk ratio should be evaluated carefully.	2C
	4.5. The repair of complex injuries (e.g., vasculo-biliary) should be delayed and not attempted intraoperatively even by expert HPB surgeons.	2C
**Antibiotic regimen**	5.1. In cases of suspected BDI during elective LC without a history of previous biliary drainage, antibiotic therapy may be considered using broad-spectrum antibiotics.	2C
	5.2. In patients with previous biliary infection (i.e., cholecystitis, cholangitis) and patients with preoperative endoscopic stenting, ENBD, or PTBD at risk of developing local and systemic sepsis, broad-spectrum antibiotics (4^th^-generation cephalosporins) are recommended, with further adjustments according to antibiograms.	1C
	5.3. In patients with biliary fistula, biloma, or bile peritonitis antibiotics should be started immediately (within 1 h) using piperacillin/tazobactam, imipenem/cilastatin, meropenem, ertapenem, or aztreonam associated with amikacin in case of shock, and using fluconazole in cases of fragile patients or delayed diagnosis.	1C
	5.4. In severe complicated intra-abdominal sepsis, open abdomen can be considered as an option for patients with organ failure and gross contamination.	2C
**Clinical, biochemical, and imaging investigations for suspected BDI**	6.1. We recommend a prompt investigation of patients who do not rapidly recover after LC, with alarm symptoms being fever, abdominal pain, distention, jaundice, nausea and vomiting (depending on the type of BDI).	2C
	6.2. The assessment of liver function tests, including serum levels of direct and indirect bilirubin, AST, ALT, ALP, GGT, and albumin, is suggested in patients with clinical signs and symptoms suggestive of BDI after LC. In critically ill patients, the serum levels of CRP, PCT, and lactate may help in the evaluation of the severity of acute inflammation and sepsis and in monitoring the response to treatment.	2C
	6.3. Abdominal triphasic CT is suggested as the first-line diagnostic imaging investigation to detect intra-abdominal fluid collections and ductal dilation. It may be complemented with the addition of CE-MRCP to obtain the exact visualization, localization and classification of BDI, which is essential for planning a tailored treatment.	2B
**Postoperatively detected BDI management**	7.1. In the case of minor BDIs (e.g., Strasberg A-D), if a drain is placed after surgery and a bile leak is noted, an observation period and nonoperative management during the first hours is an option. If no drain is placed during surgery, the percutaneous treatment of the collection with drain placement can be useful.	2C
	7.2. For minor BDIs, if no improvements or worsening of symptoms occurs during the clinical observation period after percutaneous drain placement, endoscopic management (by ERCP with biliary sphincterotomy and stent placement) becomes mandatory.	1C
	7.3. In major BDIs (e.g., Strasberg E1–E2) diagnosed in the immediate postoperative period (within 72 h), we recommend referral to a center with expertise in HPB procedures, if that expertise is locally unavailable. An urgent surgical repair with bilioenteric anastomosis Roux-en-Y hepaticojejunostomy could then be performed.	1C
	7.4. In major BDIs diagnosed between 72 h and 3 weeks, we recommend percutaneous drainage of the fluid collections whenever present, targeted antibiotics, and nutritional support. During this period, an ERCP (sphincterotomy with or without stent) can be considered to reduce the pressure gradient in the biliary tree and a PTBD could be useful for septic patients with a complete obstruction of the common bile duct. After a minimum of 3 weeks, if the patient’s general conditions allow and the acute or subacute situation is resolved (e.g., closure of the biliary fistula), the Roux-en-Y hepaticojejunostomy should be performed.	2C
	7.5. When major BDIs are recognized late after the index LC and there are clinical manifestations of stricture, Roux-en-Y hepaticojejunostomy should be performed.	2C
	7.6. When major BDIs present as diffuse biliary peritonitis, urgent abdominal cavity lavage and drainage are required as first step of treatment to achieve infection source control.	1C

The present WSES guidelines contribute to clarifying the complex decision-making process that the surgeon has to face once a BDI is suspected, detected, and diagnosed. BDI management requires not only clinical knowledge and surgical skills but also a sensible evaluation of the availability of local resources and the experience of the medical team in terms of HPB surgery. Better patient outcomes, with decreased morbidity and long-term complications, are expected when BDIs are addressed at high-volume centers by experienced multidisciplinary teams. However, efforts should be made to guarantee the best treatment options for any patient experiencing BDI, irrespective of the hospital, country, or geographical inequalities; this could be achieved by the progressive implementation of the present guidelines in clinical practice to standardize BDI detection and management among surgeons and clinicians worldwide.

## Supplementary Information

**Additional file 1.**

**Additional file 2.**

## Data Availability

There are no data from individual authors that reach the criteria for availability.

## Abbreviations

AC: Acute cholecystitis
ALP: Alkaline phosphatase
ALT: Alanine aminotransferase
AST: Aspartate aminotransferase
BDI: Bile duct injuries
CBC: Complete blood count
CI: Confidence interval
CRP: C-reactive protein
CT: Computed tomography
CVS: Critical view of safety
ENBD: Endoscopic nasobiliary drainage
ERCP: Endoscopic retrograde cholangiopancreatography
Gd-BOPTA: Gadobenate dimeglumine
Gd-EOB-DTPA: Gadoxetic acid disodium
GGT: gamma-glutamyl transpeptidase
HPB: hepato-pancreato-biliary
HR: Hazard ratio
HS: Hepatobiliary scintigraphy
ICG-C: Indocyanine green fluorescence cholangiography
IOC: Intraoperative cholangiography
IOUS: Intraoperative ultrasonography
LC: Laparoscopic cholecystectomy
MRCP: Magnetic resonance cholangiopancreatography
OA: Open abdomen
OR: Odds ratio
PCT: Procalcitonin
PTBD: Percutaneous transhepatic biliary drainage
PTC: Percutaneous transhepatic cholangiography
RCT: Randomized controlled trial
STC: Subtotal cholecystectomy
US: Ultrasonography

## References

[1] Catena F, Moore F, Ansaloni L, Leppaniemi A, Sartelli M, Peitzmann AB (2014). Emergency surgeon: “last of the mohicans” 2014-2016 editorial policy WSES- WJES: position papers, guidelines, courses, books and original research; from WJES impact factor to WSES congress impact factor. World J Emerg Surg.

[2] Ceresoli M, Coccolini F, Biffl WL, Sartelli M, Ansaloni L, Moore EE, di Saverio S, Kluger Y, Catena F (2020). WSES guidelines updates. World J Emerg Surg..

[3] Coccolini F, Kluger Y, Ansaloni L, Moore EE, Coimbra R, Fraga GP, Kirkpatrick A, Peitzman A, Maier R, Baiocchi G, Agnoletti V, Gamberini E, Leppaniemi A, Ivatury R, Sugrue M, Sartelli M, di Saverio S, Biffl W, Catena F (2018). WSES worldwide emergency general surgery formation and evaluation project. World J Emerg Surg..

[4] Alexander HC, Bartlett AS, Wells CI, Hannam JA, Moore MR, Poole GH (2018). Reporting of complications after laparoscopic cholecystectomy: a systematic review. HPB (Oxford).

[5] Brunt LM, Deziel DJ, Telem DA, Strasberg SM, Aggarwal R, Asbun H, Bonjer J, McDonald M, Alseidi A, Ujiki M, Riall TS, Hammill C, Moulton CA, Pucher PH, Parks RW, Ansari MT, Connor S, Dirks RC, Anderson B, Altieri MS, Tsamalaidze L, Stefanidis D, and the Prevention of Bile Duct Injury Consensus Work Group (2020). Safe cholecystectomy multi-society practice guideline and state of the art consensus conference on prevention of bile duct injury during cholecystectomy. Ann Surg..

[6] Kapoor VK, Kapoor V (2020). Epidemiology of bile duct injury., in post-cholecystectomy bile duct injury.

[7] Hogan NM, Dorcaratto D, Hogan AM, Nasirawan F, McEntee P, Maguire D (2016). Iatrogenic common bile duct injuries: increasing complexity in the laparoscopic era: a prospective cohort study. Int J Surg.

[8] Pulvirenti E, Toro A, Gagner M, Mannino M, Di Carlo I (2013). Increased rate of cholecystectomies performed with doubtful or no indications after laparoscopy introduction: a single center experience. BMC Surg..

[9] Fletcher R, Cortina CS, Kornfield H, Varelas A, Li R, Veenstra B, Bonomo S (2019). Bile duct injuries: a contemporary survey of surgeon attitudes and experiences. Surg Endosc.

[10] Strasberg SM, Gouma DJ (2012). ʻExtremeʼ vasculobiliary injuries: association with fundus-down cholecystectomy in severely inflamed gallbladders. HPB (Oxford).

[11] Booij KAC, de Reuver PR, van Dieren S, van Delden OM, Rauws EA, Busch OR, van Gulik TM, Gouma DJ (2018). Long-term impact of bile duct injury on morbidity, mortality, quality of life, and work related limitations. Ann Surg..

[12] Fong ZV, Pitt HA, Strasberg SM, Loehrer AP, Sicklick JK, Talamini MA, Lillemoe KD, Chang DC, California Cholecystectomy Group (2018). Diminished survival in patients with bile leak and ductal injury: management strategy and outcomes. J Am Coll Surg..

[13] Koppatz H, Sallinen V, Mäkisalo H, Nordin A (2020). Outcomes and quality of life after major bile duct injury in long-term follow-up. Surg Endosc.

[14] Cho JY, Baron TH, Carr-Locke DL, Chapman WC, Costamagna G, de Santibanes E (2018). Proposed standards for reporting outcomes of treating biliary injuries. HPB (Oxford).

[15] Iwashita Y, Hibi T, Ohyama T, Umezawa A, Takada T, Strasberg SM, Asbun HJ, Pitt HA, Han HS, Hwang TL, Suzuki K, Yoon YS, Choi IS, Yoon DS, Huang WSW, Yoshida M, Wakabayashi G, Miura F, Okamoto K, Endo I, de Santibañes E, Giménez ME, Windsor JA, Garden OJ, Gouma DJ, Cherqui D, Belli G, Dervenis C, Deziel DJ, Jonas E, Jagannath P, Supe AN, Singh H, Liau KH, Chen XP, Chan ACW, Lau WY, Fan ST, Chen MF, Kim MH, Honda G, Sugioka A, Asai K, Wada K, Mori Y, Higuchi R, Misawa T, Watanabe M, Matsumura N, Rikiyama T, Sata N, Kano N, Tokumura H, Kimura T, Kitano S, Inomata M, Hirata K, Sumiyama Y, Inui K, Yamamoto M (2017). Delphi consensus on bile duct injuries during laparoscopic cholecystectomy: an evolutionary cul-de-sac or the birth pangs of a new technical framework?. J Hepatobiliary Pancreat Sci..

[16] Chaudhary A, Manisegran M, Chandra A, Agarwal AK, Sachdev AK (2001). How do bile duct injuries sustained during laparoscopic cholecystectomy differ from those during open cholecystectomy?. J Laparoendosc Adv Surg Tech A..

[17] Bektas H, Schrem H, Winny M, Klempnauer J (2007). Surgical treatment and outcome of iatrogenic bile duct lesions after cholecystectomy and the impact of different clinical classification systems. Br J Surg..

[18] Mercado MA, Dominguez I (2011). Classification and management of bile duct injuries. World J Gastrointest Surg..

[19] de Reuver PR, Busch OR, Rauws EA, Lameris JS, van Gulik TM, Gouma DJ (2007). Long-term results of a primary end-to-end anastomosis in peroperative detected bile duct injury. J Gastrointest Surg..

[20] Lindemann J, Jonas E, Kotze U, Krige JEJ (2020). Evolution of bile duct repair in a low and middle-income country (LMIC): a comparison of diagnosis, referral, management and outcomes in repair of bile duct injury after laparoscopic cholecystectomy from 1991 to 2004 and 2005-2017. HPB (Oxford).

[21] de'Angelis, N., S. Di Saverio, O. Chiara, M. Sartelli, A. Martinez-Perez, F. Patrizi, et al., 2017 WSES guidelines for the management of iatrogenic colonoscopy perforation*.* World J Emerg Surg. 2018; 13: 5. 10.1186/s13017-018-0162-9. 10.1186/s13017-018-0162-9 PMC5784542 29416554

[22] Di Saverio S, Podda M, De Simone B, Ceresoli M, Augustin G, Gori A (2020). Diagnosis and treatment of acute appendicitis: 2020 update of the WSES Jerusalem guidelines. World J Emerg Surg..

[23] Guyatt G, Gutterman D, Baumann MH, Addrizzo-Harris D, Hylek EM, Phillips B, Raskob G, Lewis SZ, Schünemann H (2006). Grading strength of recommendations and quality of evidence in clinical guidelines: report from an American college of chest physicians task force. Chest..

[24] Brouwers MC, Kho ME, Browman GP, Burgers JS, Cluzeau F, Feder G, Fervers B, Graham ID, Grimshaw J, Hanna SE, Littlejohns P, Makarski J, Zitzelsberger L, AGREE Next Steps Consortium (2010). AGREE II: advancing guideline development, reporting and evaluation in health care. J Clin Epidemiol..

[25] Chen H, Siwo EA, Khu M, Tian Y (2018). Current trends in the management of Mirizzi syndrome: a review of literature. Medicine (Baltimore).

[26] Clemente G, Tringali A, De Rose AM, Panettieri E, Murazio M, Nuzzo G (2018). Mirizzi syndrome: diagnosis and management of a challenging biliary disease. Can J Gastroenterol Hepatol..

[27] Senra F, Navaratne L, Acosta A, Martínez-Isla A (2020). Laparoscopic management of type II Mirizzi syndrome. Surg Endosc..

[28] Coccolini F, Catena F, Pisano M, Gheza F, Fagiuoli S, Di Saverio S (2015). Open versus laparoscopic cholecystectomy in acute cholecystitis. Systematic review and meta-analysis. Int J Surg..

[29] Glavic Z, Begic L, Simlesa D, Rukavina A (2001). Treatment of acute cholecystitis. A comparison of open vs laparoscopic cholecystectomy. Surg Endosc..

[30] Unger SW, Rosenbaum G, Unger HM, Edelman DS (1993). A comparison of laparoscopic and open treatment of acute cholecystitis. Surg Endosc..

[31] Catena F, Ansaloni L, Bianchi E, Di Saverio S, Coccolini F, Vallicelli C (2013). The ACTIVE (Acute Cholecystitis Trial Invasive Versus Endoscopic) study: multicenter randomized, double-blind, controlled trial of laparoscopic versus open surgery for acute cholecystitis. Hepatogastroenterology..

[32] Boo YJ, Kim WB, Kim J, Song TJ, Choi SY, Kim YC (2007). Systemic immune response after open versus laparoscopic cholecystectomy in acute cholecystitis: a prospective randomized study. Scand J Clin Lab Invest..

[33] Johansson M, Thune A, Nelvin L, Stiernstam M, Westman B, Lundell L (2005). Randomized clinical trial of open versus laparoscopic cholecystectomy in the treatment of acute cholecystitis. Br J Surg..

[34] Kiviluoto T, Sirén J, Luukkonen P, Kivilaakso E (1998). Randomised trial of laparoscopic versus open cholecystectomy for acute and gangrenous cholecystitis. Lancet..

[35] Eldar S, Sabo E, Nash E, Abrahamson J, Matter I (1998). Laparoscopic cholecystectomy for the various types of gallbladder inflammation: a prospective trial. Surg Laparosc Endosc..

[36] Tang B, Cuschieri A (2006). Conversions during laparoscopic cholecystectomy: risk factors and effects on patient outcome. J Gastrointest Surg..

[37] Halachmi S, DiCastro N, Matter I, Cohen A, Sabo E, Mogilner JG (2000). Laparoscopic cholecystectomy for acute cholecystitis: how do fever and leucocytosis relate to conversion and complications?. Eur J Surg..

[38] Giger U, Michel JM, Vonlanthen R, Becker K, Kocher T, Krähenbühl L (2005). Laparoscopic cholecystectomy in acute cholecystitis: indication, technique, risk and outcome. Langenbecks Arch Surg..

[39] Pisano M, Allievi N, Gurusamy K, Borzellino G, Cimbanassi S, Boerna D, Coccolini F, Tufo A, di Martino M, Leung J, Sartelli M, Ceresoli M, Maier RV, Poiasina E, de Angelis N, Magnone S, Fugazzola P, Paolillo C, Coimbra R, di Saverio S, de Simone B, Weber DG, Sakakushev BE, Lucianetti A, Kirkpatrick AW, Fraga GP, Wani I, Biffl WL, Chiara O, Abu-Zidan F, Moore EE, Leppäniemi A, Kluger Y, Catena F, Ansaloni L (2020). 2020 World Society of Emergency Surgery updated guidelines for the diagnosis and treatment of acute calculus cholecystitis. World J Emerg Surg..

[40] Abdalla S, Pierre S, Ellis H (2013). Calotʼs triangle. Clin Anat..

[41] Skandalakis JE, Skandalakis LJ, Skandalakis PN, Skandalakis JE, Skandalakis LJ, Skandalakis PN (2000). Extrahepatic biliary tract, in Surgical anatomy and technique.

[42] Sgaramella LI, Gurrado A, Pasculli A, de Angelis N, Memeo R, Prete FP, et al. The critical view of safety during laparoscopic cholecystectomy: Strasberg Yes or No? An Italian multicentre study. Surg Endosc. 2020. 10.1007/s00464-020-07852-6. 10.1007/s00464-020-07852-6 PMC8195809 32780231

[43] Booij KA, de Reuver PR, Nijsse B, Busch OR, van Gulik TM, Gouma DJ (2014). Insufficient safety measures reported in operation notes of complicated laparoscopic cholecystectomies. Surgery..

[44] Kohn JF, Trenk A, Kuchta K, Lapin B, Denham W, Linn JG, Haggerty S, Joehl R, Ujiki MB (2018). Characterization of common bile duct injury after laparoscopic cholecystectomy in a high-volume hospital system. Surg Endosc..

[45] Avgerinos C, Kelgiorgi D, Touloumis Z, Baltatzi L, Dervenis C (2009). One thousand laparoscopic cholecystectomies in a single surgical unit using the “critical view of safety” technique. J Gastrointest Surg..

[46] Sanjay P, Fulke JL, Exon DJ (2010). ʻCritical view of safetyʼ as an alternative to routine intraoperative cholangiography during laparoscopic cholecystectomy for acute biliary pathology. J Gastrointest Surg..

[47] Archer SB, Brown DW, Smith CD, Branum GD, Hunter JG (2001). Bile duct injury during laparoscopic cholecystectomy: results of a national survey. Ann Surg..

[48] Strasberg SM, Pucci MJ, Brunt LM, Deziel DJ (2016). Subtotal Cholecystectomy-“fenestrating” vs “reconstituting” subtypes and the prevention of bile duct injury: definition of the optimal procedure in difficult operative conditions. J Am Coll Surg..

[49] Wang YC, Yang HR, Chung PK, Jeng LB, Chen RJ (2006). Role of fundus-first laparoscopic cholecystectomy in the management of acute cholecystitis in elderly patients. J Laparoendosc Adv Surg Tech A..

[50] Rosenberg J, Leinskold T (2004). Dome down laparosonic cholecystectomy. Scand J Surg..

[51] Ichihara T, Takada M, Ajiki T, Fukumoto S, Urakawa T, Nagahata Y (2004). Tape ligature of cystic duct and fundus-down approach for safety laparoscopic cholecystectomy: outcome of 500 patients. Hepatogastroenterology..

[52] Huang SM, Hsiao KM, Pan H, Yao CC, Lai TJ, Chen LY, Wu CW, Lui WY (2011). Overcoming the difficulties in laparoscopic management of contracted gallbladders with gallstones: possible role of fundus-down approach. Surg Endosc..

[53] Gigot J, Etienne J, Aerts R, Wibin E, Dallemagne B, Deweer F (1997). The dramatic reality of biliary tract injury during laparoscopic cholecystectomy. An anonymous multicenter Belgian survey of 65 patients. Surg Endosc..

[54] Hussain A (2011). Difficult laparoscopic cholecystectomy: current evidence and strategies of management. Surg Laparosc Endosc Percutan Tech..

[55] Elshaer M, Gravante G, Thomas K, Sorge R, Al-Hamali S, Ebdewi H (2015). Subtotal cholecystectomy for “difficult gallbladders”: systematic review and meta-analysis. JAMA Surg..

[56] Sewefy AM, Hassanen AM, Atyia AM, Gaafar AM (2017). Retroinfundibular laparoscopic cholecystectomy versus standard laparoscopic cholecystectomy in difficult cases. Int J Surg..

[57] Lidsky ME, Speicher PJ, Ezekian B, Holt EW, Nussbaum DP, Castleberry AW (2017). Subtotal cholecystectomy for the hostile gallbladder: failure to control the cystic duct results in significant morbidity. HPB (Oxford).

[58] van Dijk AH, Donkervoort SC, Lameris W, de Vries E, Eijsbouts QAJ, Vrouenraets BC, Busch OR, Boermeester MA, de Reuver PR (2017). Short- and long-term outcomes after a reconstituting and fenestrating subtotal cholecystectomy. J Am Coll Surg..

[59] Chehade M, Kakala B, Sinclair JL, Pang T, Al Asady R, Richardson A (2019). Intraoperative detection of aberrant biliary anatomy via intraoperative cholangiography during laparoscopic cholecystectomy. ANZ J Surg..

[60] Ding GQ, Cai W, Qin MF (2015). Is intraoperative cholangiography necessary during laparoscopic cholecystectomy for cholelithiasis?. World J Gastroenterol..

[61] Ford JA, Soop M, Du J, Loveday BP, Rodgers M (2012). Systematic review of intraoperative cholangiography in cholecystectomy. Br J Surg..

[62] Goldstein SD, Lautz TB (2020). Fluorescent cholangiography during laparoscopic cholecystectomy: shedding new light on biliary anatomy. JAMA Surg.

[63] Osayi SN, Wendling MR, Drosdeck JM, Chaudhry UI, Perry KA, Noria SF, Mikami DJ, Needleman BJ, Muscarella P, Abdel-Rasoul M, Renton DB, Melvin WS, Hazey JW, Narula VK (2015). Near-infrared fluorescent cholangiography facilitates identification of biliary anatomy during laparoscopic cholecystectomy. Surg Endosc..

[64] Pesce A, Piccolo G, La Greca G, Puleo S (2015). Utility of fluorescent cholangiography during laparoscopic cholecystectomy: a systematic review. World J Gastroenterol..

[65] Prevot F, Rebibo L, Cosse C, Browet F, Sabbagh C, Regimbeau JM (2014). Effectiveness of intraoperative cholangiography using indocyanine green (versus contrast fluid) for the correct assessment of extrahepatic bile ducts during day-case laparoscopic cholecystectomy. J Gastrointest Surg..

[66] Cao AM, Eslick GD, Cox MR (2015). Early Cholecystectomy is superior to delayed cholecystectomy for acute cholecystitis: a meta-analysis. J Gastrointest Surg..

[67] Tornqvist B, Waage A, Zheng Z, Ye W, Nilsson M (2016). Severity of acute cholecystitis and risk of iatrogenic bile duct injury during cholecystectomy, a population-based case-control study. World J Surg..

[68] de Mestral C, Rotstein OD, Laupacis A, Hoch JS, Zagorski B, Alali AS, Nathens AB (2014). Comparative operative outcomes of early and delayed cholecystectomy for acute cholecystitis: a population-based propensity score analysis. Ann Surg..

[69] Brooks KR, Scarborough JE, Vaslef SN, Shapiro ML (2013). No need to wait: an analysis of the timing of cholecystectomy during admission for acute cholecystitis using the American College of Surgeons National Surgical Quality Improvement Program database. J Trauma Acute Care Surg..

[70] Johner A, Raymakers A, Wiseman SM (2013). Cost utility of early versus delayed laparoscopic cholecystectomy for acute cholecystitis. Surg Endosc..

[71] Zafar SN, Obirieze A, Adesibikan B, Cornwell EE, Fullum TM, Tran DD (2015). Optimal time for early laparoscopic cholecystectomy for acute cholecystitis. JAMA Surg.

[72] Pourhoseingholi MA, Vahedi M, Rahimzadeh M (2013). Sample size calculation in medical studies. Gastroenterol Hepatol Bed Bench..

[73] Harboe KM, Bardram L (2011). Nationwide quality improvement of cholecystectomy: results from a national database. Int J Qual Health Care..

[74] El-Dhuwaib Y, Slavin J, Corless DJ, Begaj I, Durkin D, Deakin M (2016). Bile duct reconstruction following laparoscopic cholecystectomy in England. Surg Endosc..

[75] Harrison VL, Dolan JP, Pham TH, Diggs BS, Greenstein AJ, Sheppard BC, Hunter JG (2011). Bile duct injury after laparoscopic cholecystectomy in hospitals with and without surgical residency programs: is there a difference?. Surg Endosc..

[76] Chuang KI, Corley D, Postlethwaite DA, Merchant M, Harris HW (2012). Does increased experience with laparoscopic cholecystectomy yield more complex bile duct injuries?. Am J Surg..

[77] Lilley EJ, Scott JW, Jiang W, Krasnova A, Raol N, Changoor N, Salim A, Haider AH, Weissman JS, Schneider EB, Cooper Z (2017). Intraoperative cholangiography during cholecystectomy among hospitalized Medicare beneficiaries with non-neoplastic biliary disease. Am J Surg..

[78] Tornqvist B, Stromberg C, Akre O, Enochsson L, Nilsson M (2015). Selective intraoperative cholangiography and risk of bile duct injury during cholecystectomy. Br J Surg..

[79] Rystedt J, Lindell G, Montgomery A (2016). Bile duct injuries associated with 55,134 cholecystectomies: treatment and outcome from a national perspective. World J Surg..

[80] Blohm M, Osterberg J, Sandblom G, Lundell L, Hedberg M, Enochsson L (2017). The sooner, the better? The importance of optimal timing of cholecystectomy in acute cholecystitis: data from the National Swedish Registry for Gallstone Surgery, GallRiks. J Gastrointest Surg..

[81] Giger U, Ouaissi M, Schmitz SF, Krahenbuhl S, Krahenbuhl L (2011). Bile duct injury and use of cholangiography during laparoscopic cholecystectomy. Br J Surg..

[82] Schwaitzberg SD, Scott DJ, Jones DB, McKinley SK, Castrillion J, Hunter TD, Michael Brunt L (2014). Threefold increased bile duct injury rate is associated with less surgeon experience in an insurance claims database: more rigorous training in biliary surgery may be needed. Surg Endosc..

[83] Fullum TM, Downing SR, Ortega G, Chang DC, Oyetunji TA, Van Kirk K (2013). Is laparoscopy a risk factor for bile duct injury during cholecystectomy?. JSLS..

[84] Mangieri CW, Hendren BP, Strode MA, Bandera BC, Faler BJ (2019). Bile duct injuries (BDI) in the advanced laparoscopic cholecystectomy era. Surg Endosc..

[85] Halbert C, Pagkratis S, Yang J, Meng Z, Altieri MS, Parikh P, Pryor A, Talamini M, Telem DA (2016). Beyond the learning curve: incidence of bile duct injuries following laparoscopic cholecystectomy normalize to open in the modern era. Surg Endosc..

[86] O'Brien S, Wei D, Bhutiani N, Rao MK, Johnston SS, Patkar A (2020). Adverse outcomes and short-term cost implications of bile duct injury during cholecystectomy. Surg Endosc..

[87] Pucher PH, Brunt LM, Davies N, Linsk A, Munshi A, Rodriguez HA (2018). Outcome trends and safety measures after 30 years of laparoscopic cholecystectomy: a systematic review and pooled data analysis. Surg Endosc..

[88] Barrett M, Asbun HJ, Chien HL, Brunt LM, Telem DA (2018). Bile duct injury and morbidity following cholecystectomy: a need for improvement. Surg Endosc..

[89] Gurusamy KS, Davidson C, Gluud C, Davidson BR. Early versus delayed laparoscopic cholecystectomy for people with acute cholecystitis. Cochrane Database Syst Rev. 2013:CD005440. 10.1002/14651858.CD005440.pub3. 10.1002/14651858.CD005440.pub3 23813477

[90] Strasberg SM, Hertl M, Soper NJ (1995). An analysis of the problem of biliary injury during laparoscopic cholecystectomy. J Am Coll Surg..

[91] Chun K (2014). Recent classifications of the common bile duct injury. Korean J Hepatobiliary Pancreat Surg.

[92] Bismuth H, Majno PE (2001). Biliary strictures: classification based on the principles of surgical treatment. World J Surg..

[93] Bismuth H, Blumbart LH (1982). Postoperative strictures of the bile duct. The biliary tract. Clinical Surgery International.

[94] McMahon, A.J., G. Fullarton, J.N. Baxter, and P.J. ʻOʼDwyer, Bile duct injury and bile leakage in laparoscopic cholecystectomy*.* Br J Surg. 1995; 82: 307-313. 10.1002/bjs.1800820308. 10.1002/bjs.1800820308 7795992

[95] Bergman JJ, van den Brink GR, Rauws EA, de Wit L, Obertop H, Huibregtse K, Tytgat GN, Gouma DJ (1996). Treatment of bile duct lesions after laparoscopic cholecystectomy. Gut..

[96] Neuhaus P, Schmidt SC, Hintze RE, Adler A, Veltzke W, Raakow R (2000). Classification and treatment of bile duct injuries after laparoscopic cholecystectomy. Chirurg..

[97] Csendes A, Navarrete C, Burdiles P, Yarmuch J (2001). Treatment of common bile duct injuries during laparoscopic cholecystectomy: endoscopic and surgical management. World J Surg..

[98] Stewart, L., C.O. Domingez, and L.W. Way, Bile duct injuries during laparoscopic cholecystectomy: a sensemaking analysis of operative reports. Proceedings of the Eighth International NDM Conference (Eds. K. Mosier & U. Fischer), Pacific Grove, CA; 2007.

[99] Lau WY, Lai EC (2007). Classification of iatrogenic bile duct injury. Hepatobiliary Pancreat Dis Int..

[100] Fingerhut A, Dziri C, Garden OJ, Gouma D, Millat B, Neugebauer E, Paganini A, Targarona E (2013). ATOM, the all-inclusive, nominal EAES classification of bile duct injuries during cholecystectomy. Surg Endosc..

[101] Kapoor VK, Kapoor VK (2020). Nomenclature and classification of bile duct injury, in post-cholecystectomy bile duct injury.

[102] Pekolj J, Alvarez FA, Palavecino M, Sanchez Claria R, Mazza O, de Santibanes E (2013). Intraoperative management and repair of bile duct injuries sustained during 10,123 laparoscopic cholecystectomies in a high-volume referral center. J Am Coll Surg..

[103] Stewart L (2014). Iatrogenic biliary injuries: identification, classification, and management. Surg Clin North Am..

[104] Quaresima S, Balla A, Palmieri L, Seitaj A, Fingerhut A, Ursi P, Paganini AM (2019). Routine near infra-red indocyanine green fluorescent cholangiography versus intraoperative cholangiography during laparoscopic cholecystectomy: a case-matched comparison. Surg Endosc.

[105] Pesce A, Palmucci S, La Greca G, Puleo S (2019). Iatrogenic bile duct injury: impact and management challenges. Clin Exp Gastroenterol..

[106] Way LW, Stewart L, Gantert W, Liu K, Lee CM, Whang K, Hunter JG (2003). Causes and prevention of laparoscopic bile duct injuries: analysis of 252 cases from a human factors and cognitive psychology perspective. Ann Surg..

[107] Bergstrom H, Larsson LG, Stenberg E (2018). Audio-video recording during laparoscopic surgery reduces irrelevant conversation between surgeons: a cohort study. BMC Surg..

[108] Prigoff JG, Sherwin M, Divino CM (2016). Ethical Recommendations for Video Recording in the Operating Room. Ann Surg..

[109] Makary MA, Xu T, Pawlik TM (2015). Can video recording revolutionise medical quality?. BMJ..

[110] Ardiles V, McCormack L, Quinonez E, Goldaracena N, Mattera J, Pekolj J (2011). Experience using liver transplantation for the treatment of severe bile duct injuries over 20 years in Argentina: results from a national survey. HPB (Oxford)..

[111] Iannelli A, Paineau J, Hamy A, Schneck AS, Schaaf C, Gugenheim J (2013). Primary versus delayed repair for bile duct injuries sustained during cholecystectomy: results of a survey of the Association Francaise de Chirurgie. HPB (Oxford).

[112] Abbasoglu O, Tekant Y, Alper A, Aydin U, Balik A, Bostanci B, Coker A, Doganay M, Gundogdu H, Hamaloglu E, Kapan M, Karademir S, Karayalcin K, Kilicturgay S, Sare M, Tumer AR, Yagci G (2016). Prevention and acute management of biliary injuries during laparoscopic cholecystectomy: expert consensus statement. Ulus Cerrahi Derg..

[113] Pucher PH, Brunt LM, Fanelli RD, Asbun HJ, Aggarwal R (2015). SAGES expert Delphi consensus: critical factors for safe surgical practice in laparoscopic cholecystectomy. Surg Endosc..

[114] Khadra H, Johnson H, Crowther J, McClaren P, Darden M, Parker G, Buell JF (2019). Bile duct injury repairs: progressive outcomes in a tertiary referral center. Surgery..

[115] Sirinek KR, Schwesinger WH (2015). Has intraoperative cholangiography during laparoscopic cholecystectomy become obsolete in the era of preoperative endoscopic retrograde and magnetic resonance cholangiopancreatography?. J Am Coll Surg..

[116] Sajid MS, Leaver C, Haider Z, Worthington T, Karanjia N, Singh KK (2012). Routine on-table cholangiography during cholecystectomy: a systematic review. Ann R Coll Surg Engl..

[117] Alves A, Farges O, Nicolet J, Watrin T, Sauvanet A, Belghiti J (2003). Incidence and consequence of an hepatic artery injury in patients with postcholecystectomy bile duct strictures. Ann Surg..

[118] Vlek SL, van Dam DA, Rubinstein SM, de Lange-de Klerk ESM, Schoonmade LJ, Tuynman JB, Meijerink WJHJ, Ankersmit M (2017). Biliary tract visualization using near-infrared imaging with indocyanine green during laparoscopic cholecystectomy: results of a systematic review. Surg Endosc..

[119] Kapoor VK (2019). ʻColleaguographyʼ in place of cholangiography, to prevent bile duct injury during laparoscopic cholecystectomy. J Minim Access Surg..

[120] Krige JE, Bornman PC, Kahn D (2010). Bile leaks and sepsis: drain now, fix later. Arch Surg..

[121] Gazzaniga GM, Filauro M, Mori L (2001). Surgical treatment of iatrogenic lesions of the proximal common bile duct. World J Surg..

[122] Silva MA, Coldham C, Mayer AD, Bramhall SR, Buckels JA, Mirza DF (2008). Specialist outreach service for on-table repair of iatrogenic bile duct injuries--a new kind of ʻtravelling surgeonʼ. Ann R Coll Surg Engl..

[123] Mourad MM, Liossis C, Gunson BK, Mergental H, Isaac J, Muiesan P, Mirza DF, Perera MTPR, Bramhall SR (2014). Etiology and management of hepatic artery thrombosis after adult liver transplantation. Liver Transpl..

[124] Fatima J, Barton JG, Grotz TE, Geng Z, Harmsen WS, Huebner M, Baron TH, Kendrick ML, Donohue JH, Que FG, Nagorney DM, Farnell MB (2010). Is there a role for endoscopic therapy as a definitive treatment for post-laparoscopic bile duct injuries?. J Am Coll Surg..

[125] Otto W, Sierdzinski J, Smaga J, Dudek K, Zieniewicz K (2018). Long-term effects and quality of life following definitive bile duct reconstruction. Medicine (Baltimore).

[126] Perera MT, Silva MA, Hegab B, Muralidharan V, Bramhall SR, Mayer AD (2011). Specialist early and immediate repair of post-laparoscopic cholecystectomy bile duct injuries is associated with an improved long-term outcome. Ann Surg..

[127] Ray S, Sanyal S, Das S, Jana K, Das AK, Khamrui S (2020). Outcomes of surgery for post-cholecystectomy bile duct injuries: an audit from a tertiary referral center. J Visc Surg..

[128] Sahajpal AK, Chow SC, Dixon E, Greig PD, Gallinger S, Wei AC (2010). Bile duct injuries associated with laparoscopic cholecystectomy: timing of repair and long-term outcomes. Arch Surg..

[129] Mesleh MG, Asbun HJ, Asbun HJ (2020). Management of common bile duct injury. The SAGES manual of biliary surgery.

[130] Wang X, Yu WL, Fu XH, Zhu B, Zhao T, Zhang YJ (2020). Early versus delayed surgical repair and referral for patients with bile duct injury: a systematic review and meta-analysis. Ann Surg..

[131] group, A.E.-A.H.A.R.C.S.m. and C. Other members of the European-African HepatoPancreatoBiliary Association Research, post cholecystectomy bile duct injury: early, intermediate or late repair with hepaticojejunostomy - an E-AHPBA multi-center study*.* HPB (Oxford). 2019; 21: 1641-1647. 10.1016/j.hpb.2019.04.003. 10.1016/j.hpb.2019.04.003 31151812

[132] Pulitano C, Parks RW, Ireland H, Wigmore SJ, Garden OJ (2011). Impact of concomitant arterial injury on the outcome of laparoscopic bile duct injury. Am J Surg..

[133] Strasberg SM, Helton WS (2011). An analytical review of vasculobiliary injury in laparoscopic and open cholecystectomy. HPB (Oxford).

[134] Truant S, Boleslawski E, Lebuffe G, Sergent G, Pruvot FR (2010). Hepatic resection for post-cholecystectomy bile duct injuries: a literature review. HPB (Oxford).

[135] De Simone B, Sartelli M, Coccolini F, Ball CG, Brambillasca P, Chiarugi M (2020). Intraoperative surgical site infection control and prevention: a position paper and future addendum to WSES intra-abdominal infections guidelines. World J Emerg Surg.

[136] Societe francaise d'anesthesie et de, r., [Antibioprophylaxis in surgery and interventional medicine (adult patients). Actualization 2010]*.* Ann Fr Anesth Reanim. 2011; 30: 168-190. 10.1016/j.annfar.2010.05.012. 10.1016/j.annfar.2010.05.012 21324634

[137] Societe francaise d'anesthesie et de, r., [Antibioprophylaxis in surgery and interventional medicine (adult patients). Actualization 2010]*.* Ann Fr Anesth Reanim. 2017. 10.1016/j.annfar.2010.05.012 21324634

[138] Venkatesan, A.M., S. Kundu, D. Sacks, M.J. Wallace, J.C. Wojak, S.C. Rose, et al., Practice guidelines for adult antibiotic prophylaxis during vascular and interventional radiology procedures. Written by the Standards of Practice Committee for the Society of Interventional Radiology and Endorsed by the Cardiovascular Interventional Radiological Society of Europe and Canadian Interventional Radiology Association [corrected]*.* J Vasc Interv Radiol. 2010; 21: 1611-1630; quiz 1631. 10.1016/j.jvir.2010.07.018 10.1016/j.jvir.2010.07.018 21029949

[139] Bratzler DW, Dellinger EP, Olsen KM, Perl TM, Auwaerter PG, Bolon MK, Fish DN, Napolitano LM, Sawyer RG, Slain D, Steinberg JP, Weinstein RA, American Society of Health-System Pharmacists, Infectious Disease Society of America, Surgical Infection Society, Society for Healthcare Epidemiology of America (2013). Clinical practice guidelines for antimicrobial prophylaxis in surgery. Am J Health Syst Pharm..

[140] Sartelli M, Chichom-Mefire A, Labricciosa FM, Hardcastle T, Abu-Zidan FM, Adesunkanmi AK, Ansaloni L, Bala M, Balogh ZJ, Beltrán MA, Ben-Ishay O, Biffl WL, Birindelli A, Cainzos MA, Catalini G, Ceresoli M, Che Jusoh A, Chiara O, Coccolini F, Coimbra R, Cortese F, Demetrashvili Z, di Saverio S, Diaz JJ, Egiev VN, Ferrada P, Fraga GP, Ghnnam WM, Lee JG, Gomes CA, Hecker A, Herzog T, Kim JI, Inaba K, Isik A, Karamarkovic A, Kashuk J, Khokha V, Kirkpatrick AW, Kluger Y, Koike K, Kong VY, Leppaniemi A, Machain GM, Maier RV, Marwah S, McFarlane ME, Montori G, Moore EE, Negoi I, Olaoye I, Omari AH, Ordonez CA, Pereira BM, Pereira Júnior GA, Pupelis G, Reis T, Sakakushev B, Sato N, Segovia Lohse HA, Shelat VG, Søreide K, Uhl W, Ulrych J, van Goor H, Velmahos GC, Yuan KC, Wani I, Weber DG, Zachariah SK, Catena F (2017). The management of intra-abdominal infections from a global perspective: 2017 WSES guidelines for management of intra-abdominal infections. World J Emerg Surg..

[141] Ansaloni L, Pisano M, Coccolini F, Peitzmann AB, Fingerhut A, Catena F, Agresta F, Allegri A, Bailey I, Balogh ZJ, Bendinelli C, Biffl W, Bonavina L, Borzellino G, Brunetti F, Burlew CC, Camapanelli G, Campanile FC, Ceresoli M, Chiara O, Civil I, Coimbra R, de Moya M, di Saverio S, Fraga GP, Gupta S, Kashuk J, Kelly MD, Khokha V, Jeekel H, Latifi R, Leppaniemi A, Maier RV, Marzi I, Moore F, Piazzalunga D, Sakakushev B, Sartelli M, Scalea T, Stahel PF, Taviloglu K, Tugnoli G, Uraneus S, Velmahos GC, Wani I, Weber DG, Viale P, Sugrue M, Ivatury R, Kluger Y, Gurusamy KS, Moore EE (2016). 2016 WSES guidelines on acute calculous cholecystitis. World J Emerg Surg..

[142] Levy MM, Evans LE, Rhodes A (2018). The Surviving Sepsis Campaign Bundle: 2018 update. Intensive Care Med..

[143] Regimbeau JM, Fuks D, Pautrat K, Mauvais F, Haccart V, Msika S, Mathonnet M, Scotté M, Paquet JC, Vons C, Sielezneff I, Millat B, Chiche L, Dupont H, Duhaut P, Cossé C, Diouf M, Pocard M, FRENCH Study Group (2014). Effect of postoperative antibiotic administration on postoperative infection following cholecystectomy for acute calculous cholecystitis: a randomized clinical trial. JAMA..

[144] Okamoto K, Suzuki K, Takada T, Strasberg SM, Asbun HJ, Endo I, Iwashita Y, Hibi T, Pitt HA, Umezawa A, Asai K, Han HS, Hwang TL, Mori Y, Yoon YS, Huang WSW, Belli G, Dervenis C, Yokoe M, Kiriyama S, Itoi T, Jagannath P, Garden OJ, Miura F, Nakamura M, Horiguchi A, Wakabayashi G, Cherqui D, de Santibañes E, Shikata S, Noguchi Y, Ukai T, Higuchi R, Wada K, Honda G, Supe AN, Yoshida M, Mayumi T, Gouma DJ, Deziel DJ, Liau KH, Chen MF, Shibao K, Liu KH, Su CH, Chan ACW, Yoon DS, Choi IS, Jonas E, Chen XP, Fan ST, Ker CG, Giménez ME, Kitano S, Inomata M, Hirata K, Inui K, Sumiyama Y, Yamamoto M (2018). Tokyo Guidelines 2018: flowchart for the management of acute cholecystitis. J Hepatobiliary Pancreat Sci..

[145] Overby DW, Apelgren KN, Richardson W, Fanelli R, G. Society of American, and S (2010). Endoscopic, SAGES guidelines for the clinical application of laparoscopic biliary tract surgery. Surg Endosc.

[146] Saad N, Darcy M (2008). Iatrogenic bile duct injury during laparoscopic cholecystectomy. Tech Vasc Interv Radiol..

[147] Coccolini F, Montori G, Ceresoli M, Catena F, Moore EE, Ivatury R (2017). The role of open abdomen in non-trauma patient: WSES consensus paper. World J Emerg Surg.

[148] Coccolini F, Roberts D, Ansaloni L, Ivatury R, Gamberini E, Kluger Y, Moore EE, Coimbra R, Kirkpatrick AW, Pereira BM, Montori G, Ceresoli M, Abu-Zidan FM, Sartelli M, Velmahos G, Fraga GP, Leppaniemi A, Tolonen M, Galante J, Razek T, Maier R, Bala M, Sakakushev B, Khokha V, Malbrain M, Agnoletti V, Peitzman A, Demetrashvili Z, Sugrue M, di Saverio S, Martzi I, Soreide K, Biffl W, Ferrada P, Parry N, Montravers P, Melotti RM, Salvetti F, Valetti TM, Scalea T, Chiara O, Cimbanassi S, Kashuk JL, Larrea M, Hernandez JAM, Lin HF, Chirica M, Arvieux C, Bing C, Horer T, de Simone B, Masiakos P, Reva V, DeAngelis N, Kike K, Balogh ZJ, Fugazzola P, Tomasoni M, Latifi R, Naidoo N, Weber D, Handolin L, Inaba K, Hecker A, Kuo-Ching Y, Ordoñez CA, Rizoli S, Gomes CA, de Moya M, Wani I, Mefire AC, Boffard K, Napolitano L, Catena F (2018). The open abdomen in trauma and non-trauma patients: WSES guidelines. World J Emerg Surg..

[149] Kirkpatrick AW, Coccolini F, Ansaloni L, Roberts DJ, Tolonen M, McKee JL (2018). Closed Or Open after Source Control Laparotomy for Severe Complicated Intra-Abdominal Sepsis (the COOL trial): study protocol for a randomized controlled trial. World J Emerg Surg..

[150] Ibrarullah M, Sankar S, Sreenivasan K, Gavini SR (2015). Management of bile duct injury at various stages of presentation: experience from a tertiary care centre. Indian J Surg..

[151] Lillemoe KD (1997). Benign post-operative bile duct strictures. Baillieres Clin Gastroenterol..

[152] Kogure H, Tsujino T, Yamamoto K, Mizuno S, Yashima Y, Yagioka H, Kawakubo K, Sasaki T, Nakai Y, Hirano K, Sasahira N, Isayama H, Tada M, Kawabe T, Omata M, Harada S, Ota Y, Koike K (2011). Fever-based antibiotic therapy for acute cholangitis following successful endoscopic biliary drainage. J Gastroenterol..

[153] van Lent AU, Bartelsman JF, Tytgat GN, Speelman P, Prins JM (2002). Duration of antibiotic therapy for cholangitis after successful endoscopic drainage of the biliary tract. Gastrointest Endosc..

[154] Jablonska B, Lampe P (2009). Iatrogenic bile duct injuries: etiology, diagnosis and management. World J Gastroenterol..

[155] Gupta V, Jayaraman S (2017). Role for laparoscopy in the management of bile duct injuries. Can J Surg..

[156] Wu JS, Peng C, Mao XH, Lv P (2007). Bile duct injuries associated with laparoscopic and open cholecystectomy: sixteen-year experience. World J Gastroenterol..

[157] Linhares BL, Magalhaes Ada G, Cardoso PM, Linhares Filho JP, Pinho JE, Costa ML (2011). Bile duct injury following cholecystectomy. Rev Col Bras Cir..

[158] Hariharan D, Psaltis E, Scholefield JH, Lobo DN (2017). Quality of life and medico-legal implications following iatrogenic bile duct injuries. World J Surg..

[159] Ahmad NZ (2011). Routine testing of liver function before and after elective laparoscopic cholecystectomy: is it necessary?. JSLS..

[160] Sakorafas G, Anagnostopoulos G, Stafyla V, Koletis T, Kotsifopoulos N, Tsiakos S (2005). Elevation of serum liver enzymes after laparoscopic cholecystectomy. N Z Med J..

[161] Ben-Ishay O, Zeltser M, Kluger Y (2017). Utility of routine blood tests after elective laparoscopic cholecystectomy for symptomatic gallstones. World J Gastrointest Surg..

[162] Davidoff AM, Branum GD, Meyers WC (1993). Clinical features and mechanisms of major laparoscopic biliary injury. Semin Ultrasound CT MR..

[163] Faix JD (2013). Biomarkers of sepsis. Crit Rev Clin Lab Sci..

[164] Fan SL, Miller NS, Lee J, Remick DG (2016). Diagnosing sepsis - The role of laboratory medicine. Clin Chim Acta..

[165] Wigham A, Alexander Grant L (2013). Radiologic assessment of hepatobiliary surgical complications. Semin Ultrasound CT MR..

[166] Laurent V, Ayav A, Hoeffel C, Bruot O, Ganne PA, Mathias J (2009). Imaging of the postoperative biliary tract. J Radiol..

[167] Lee CM, Stewart L, Way LW (2000). Postcholecystectomy abdominal bile collections. Arch Surg..

[168] Mule S, Colosio A, Cazejust J, Kianmanesh R, Soyer P, Hoeffel C (2015). Imaging of the postoperative liver: review of normal appearances and common complications. Abdom Imaging..

[169] Mungai F, Berti V, Colagrande S (2013). Bile leak after elective laparoscopic cholecystectomy: role of MR imaging. J Radiol Case Rep..

[170] Tripathi M, Chandrashekar N, Kumar R, Thomas EJ, Agarwal S, Bal CS (2004). Hepatobiliary scintigraphy. An effective tool in the management of bile leak following laparoscopic cholecystectomy. Clin Imaging.

[171] Trerotola SO, Savader SJ, Lund GB, Venbrux AC, Sostre S, Lillemoe KD, Cameron JL, Osterman FA (1992). Biliary tract complications following laparoscopic cholecystectomy: imaging and intervention. Radiology..

[172] Barkun AN, Rezieg M, Mehta SN, Pavone E, Landry S, Barkun JS, Fried GM, Bret P, Cohen A (1997). Postcholecystectomy biliary leaks in the laparoscopic era: risk factors, presentation, and management. McGill Gallstone Treatment Group. Gastrointest Endosc..

[173] McGahan JP, Stein M (1995). Complications of laparoscopic cholecystectomy: imaging and intervention. AJR Am J Roentgenol..

[174] Fidelman N, Kerlan RK, Laberge JM, Gordon RL (2011). Accuracy of percutaneous transhepatic cholangiography in predicting the location and nature of major bile duct injuries. J Vasc Interv Radiol..

[175] Lindemann J, Kloppers C, Burmeister S, Bernon M, Jonas E (2019). Mind the gap! Extraluminal percutaneous-endoscopic rendezvous with a self-expanding metal stent for restoring continuity in major bile duct injury: a case series. Int J Surg Case Rep..

[176] Schreuder AM, Booij KAC, de Reuver PR, van Delden OM, van Lienden KP, Besselink MG (2018). Percutaneous-endoscopic rendezvous procedure for the management of bile duct injuries after cholecystectomy: short- and long-term outcomes. Endoscopy..

[177] Freeman ML, DiSario JA, Nelson DB, Fennerty MB, Lee JG, Bjorkman DJ, Overby CS, Aas J, Ryan ME, Bochna GS, Shaw MJ, Snady HW, Erickson RV, Moore JP, Roel JP (2001). Risk factors for post-ERCP pancreatitis: a prospective, multicenter study. Gastrointest Endosc..

[178] Matsubayashi H, Fukutomi A, Kanemoto H, Maeda A, Matsunaga K, Uesaka K (2009). Risk of pancreatitis after endoscopic retrograde cholangiopancreatography and endoscopic biliary drainage. HPB (Oxford)..

[179] Aduna M, Larena JA, Martin D, Martinez-Guerenu B, Aguirre I, Astigarraga E (2005). Bile duct leaks after laparoscopic cholecystectomy: value of contrast-enhanced MRCP. Abdom Imaging..

[180] Park MS, Kim KW, Yu JS, Kim MJ, Kim KW, Lim JS, Cho ES, Yoon DS, Kim TK, Lee SI, Lee JD, Lee WJ, Ha HK, Lee JT, Yoo HS (2004). Early biliary complications of laparoscopic cholecystectomy: evaluation on T2-weighted MR cholangiography in conjunction with mangafodipir trisodium-enhanced 3D T1-weighted MR cholangiography. AJR Am J Roentgenol..

[181] An SK, Lee JM, Suh KS, Lee NJ, Kim SH, Kim YJ, Han JK, Choi BI (2006). Gadobenate dimeglumine-enhanced liver MRI as the sole preoperative imaging technique: a prospective study of living liver donors. AJR Am J Roentgenol..

[182] Cieszanowski A, Stadnik A, Lezak A, Maj E, Zieniewicz K, Rowinska-Berman K, Grudzinski IP, Krawczyk M, Rowiński O (2013). Detection of active bile leak with Gd-EOB-DTPA enhanced MR cholangiography: comparison of 20-25 min delayed and 60-180 min delayed images. Eur J Radiol..

[183] Kantarci M, Pirimoglu B, Karabulut N, Bayraktutan U, Ogul H, Ozturk G (2013). Non-invasive detection of biliary leaks using Gd-EOB-DTPA-enhanced MR cholangiography: comparison with T2-weighted MR cholangiography. Eur Radiol..

[184] Lee NK, Kim S, Lee JW, Lee SH, Kang DH, Kim GH, Seo HI (2009). Biliary MR imaging with Gd-EOB-DTPA and its clinical applications. Radiographics..

[185] Ribeiro BJ, Alves AMA, de Oliveira RS, Velloni F, D'Ippolito G (2019). The role of gadoxetic acid-enhanced magnetic resonance cholangiography in the evaluation of postoperative bile duct injury: pictorial essay. Radiol Bras.

[186] Wong YC, Wang LJ, Wu CH, Chen HW, Fu CJ, Yuan KC, Lin BC, Hsu YP, Kang SC (2018). Detection and characterization of traumatic bile leaks using Gd-EOB-DTPA enhanced magnetic resonance cholangiography. Sci Rep..

[187] Boraschi P, Donati F, Pacciardi F, Ghinolfi D, Falaschi F (2018). Biliary complications after liver transplantation: assessment with MR cholangiopancreatography and MR imaging at 3T device. Eur J Radiol..

[188] Boerma D, Rauws EA, Keulemans YC, Bergman JJ, Obertop H, Huibregtse K (2001). Impaired quality of life 5 years after bile duct injury during laparoscopic cholecystectomy: a prospective analysis. Ann Surg..

[189] Flum DR, Cheadle A, Prela C, Dellinger EP, Chan L (2003). Bile duct injury during cholecystectomy and survival in medicare beneficiaries. JAMA..

[190] Nuzzo, G., F. Giuliante, I. Giovannini, M. Murazio, F. DʼAcapito, F. Ardito, et al., Advantages of multidisciplinary management of bile duct injuries occurring during cholecystectomy*.* Am J Surg. 2008; 195: 763-769. 10.1016/j.amjsurg.2007.05.046, 6. 10.1016/j.amjsurg.2007.05.046 18367147

[191] Kimura T, Suzuki K, Umehara Y, Kawabe A, Wada H (2005). Features and management of bile leaks after laparoscopic cholecystectomy. J Hepatobiliary Pancreat Surg..

[192] Ogura T, Masuda D, Saori O, Wataru T, Sano T, Okuda A, Miyano A, Kitano M, Abdel-aal UM, Takeuchi T, Fukunishi S, Higuchi K (2016). Clinical outcome of endoscopic ultrasound-guided liver abscess drainage using self-expandable covered metallic stent (with video). Dig Dis Sci..

[193] Zerem E, Omerovic S (2009). Minimally invasive management of biliary complications after laparoscopic cholecystectomy. Eur J Intern Med..

[194] Battal M, Yazici P, Bostanci O, Karatepe O (2019). Early surgical repair of bile duct injuries following laparoscopic cholecystectomy: the sooner the better. Surg J (N Y).

[195] Walsh RM, Vogt DP, Ponsky JL, Brown N, Mascha E, Henderson JM (2004). Management of failed biliary repairs for major bile duct injuries after laparoscopic cholecystectomy. J Am Coll Surg..

[196] Adler DG, Papachristou GI, Taylor LJ, McVay T, Birch M, Francis G, Zabolotsky A, Laique SN, Hayat U, Zhan T, Das R, Slivka A, Rabinovitz M, Munigala S, Siddiqui AA (2017). Clinical outcomes in patients with bile leaks treated via ERCP with regard to the timing of ERCP: a large multicenter study. Gastrointest Endosc..

[197] Chathadi KV, Chandrasekhara V, Acosta RD, Decker GA, Early DS, Eloubeidi MA (2015). The role of ERCP in benign diseases of the biliary tract. Gastrointest Endosc..

[198] Agarwal N, Sharma BC, Garg S, Kumar R, Sarin SK (2006). Endoscopic management of postoperative bile leaks. Hepatobiliary Pancreat Dis Int..

[199] Canena J, Horta D, Coimbra J, Meireles L, Russo P, Marques I, Ricardo L, Rodrigues C, Capela T, Carvalho D, Loureiro R, Dias AM, Ramos G, Coutinho AP, Romão C, Veiga PM (2015). Outcomes of endoscopic management of primary and refractory postcholecystectomy biliary leaks in a multicentre review of 178 patients. BMC Gastroenterol..

[200] de Reuver PR, Rauws EA, Vermeulen M, Dijkgraaf MG, Gouma DJ, Bruno MJ (2007). Endoscopic treatment of post-surgical bile duct injuries: long term outcome and predictors of success. Gut..

[201] Dumonceau JM, Tringali A, Papanikolaou IS, Blero D, Mangiavillano B, Schmidt A (2018). Endoscopic biliary stenting: indications, choice of stents, and results: European Society of Gastrointestinal Endoscopy (ESGE) Clinical Guideline - Updated October 2017. Endoscopy..

[202] Kaffes AJ, Hourigan L, De Luca N, Byth K, Williams SJ, Bourke MJ (2005). Impact of endoscopic intervention in 100 patients with suspected postcholecystectomy bile leak. Gastrointest Endosc..

[203] Sandha GS, Bourke MJ, Haber GB, Kortan PP (2004). Endoscopic therapy for bile leak based on a new classification: results in 207 patients. Gastrointest Endosc..

[204] Singh V, Singh G, Verma GR, Gupta R (2010). Endoscopic management of postcholecystectomy biliary leakage. Hepatobiliary Pancreat Dis Int..

[205] Pioche M, Ponchon T (2013). Management of bile duct leaks. J Visc Surg..

[206] Barton JR, Russell RC, Hatfield AR (1995). Management of bile leaks after laparoscopic cholecystectomy. Br J Surg..

[207] Dolay K, Soylu A, Aygun E (2010). The role of ERCP in the management of bile leakage: endoscopic sphincterotomy versus biliary stenting. J Laparoendosc Adv Surg Tech A..

[208] Mavrogiannis C, Liatsos C, Papanikolaou IS, Karagiannis S, Galanis P, Romanos A (2006). Biliary stenting alone versus biliary stenting plus sphincterotomy for the treatment of post-laparoscopic cholecystectomy biliary leaks: a prospective randomized study. Eur J Gastroenterol Hepatol..

[209] Langerth A, Sandblom G, Karlson BM (2015). Long-term risk for acute pancreatitis, cholangitis, and malignancy more than 15 years after endoscopic sphincterotomy: a population-based study. Endoscopy..

[210] Perri V, Familiari P, Tringali A, Boskoski I, Costamagna G (2011). Plastic biliary stents for benign biliary diseases. Gastrointest Endosc Clin N Am..

[211] Rustagi T, Aslanian HR (2014). Endoscopic management of biliary leaks after laparoscopic cholecystectomy. J Clin Gastroenterol..

[212] Costamagna G, Pandolfi M, Mutignani M, Spada C, Perri V (2001). Long-term results of endoscopic management of postoperative bile duct strictures with increasing numbers of stents. Gastrointest Endosc..

[213] Costamagna G, Tringali A, Perri V, Familiari P, Boškoski I, Barbaro F, Landi R (2020). Endotherapy of postcholecystectomy biliary strictures with multiple plastic stents: long-term results in a large cohort of patients. Gastrointest Endosc..

[214] Schreuder AM, Busch OR, Besselink MG, Ignatavicius P, Gulbinas A, Barauskas G, Gouma DJ, van Gulik TM (2020). Long-term impact of iatrogenic bile duct injury. Dig Surg..

[215] Canena J, Liberato M, Meireles L, Marques I, Romão C, Coutinho AP, Neves BC, Veiga PM (2015). A non-randomized study in consecutive patients with postcholecystectomy refractory biliary leaks who were managed endoscopically with the use of multiple plastic stents or fully covered self-expandable metal stents (with videos). Gastrointest Endosc..

[216] Bergman JJ, Burgemeister L, Bruno MJ, Rauws EA, Gouma DJ, Tytgat GN (2001). Long-term follow-up after biliary stent placement for postoperative bile duct stenosis. Gastrointest Endosc..

[217] Kassab C, Prat F, Liguory C, Meduri B, Ducot B, Fritsch J (2006). Endoscopic management of post-laparoscopic cholecystectomy biliary strictures. Long-term outcome in a multicenter study. Gastroenterol Clin Biol..

[218] de Jong EA, Moelker A, Leertouwer T, Spronk S, Van Dijk M, van Eijck CH (2013). Percutaneous transhepatic biliary drainage in patients with postsurgical bile leakage and nondilated intrahepatic bile ducts. Dig Surg..

[219] Eum YO, Park JK, Chun J, Lee SH, Ryu JK, Kim YT, Yoon YB, Yoon CJ, Han HS, Hwang JH (2014). Non-surgical treatment of post-surgical bile duct injury: clinical implications and outcomes. World J Gastroenterol..

[220] Dageforde LA, Landman MP, Feurer ID, Poulose B, Pinson CW, Moore DE (2012). A cost-effectiveness analysis of early vs late reconstruction of iatrogenic bile duct injuries. J Am Coll Surg..

[221] Thomson BN, Parks RW, Madhavan KK, Wigmore SJ, Garden OJ (2006). Early specialist repair of biliary injury. Br J Surg..

[222] Fischer CP, Fahy BN, Aloia TA, Bass BL, Gaber AO, Ghobrial RM (2009). Timing of referral impacts surgical outcomes in patients undergoing repair of bile duct injuries. HPB (Oxford).

[223] Deziel DJ, Millikan KW, Economou SG, Doolas A, Ko ST, Airan MC (1993). Complications of laparoscopic cholecystectomy: a national survey of 4,292 hospitals and an analysis of 77,604 cases. Am J Surg..

[224] Goykhman Y, Kory I, Small R, Kessler A, Klausner JM, Nakache R, Ben-Haim M (2008). Long-term outcome and risk factors of failure after bile duct injury repair. J Gastrointest Surg..

[225] Russell JC, Walsh SJ, Mattie AS, Lynch JT (1996). Bile duct injuries, 1989-1993. A statewide experience. Connecticut Laparoscopic Cholecystectomy Registry. Arch Surg.

[226] Stewart L, Way LW (1995). Bile duct injuries during laparoscopic cholecystectomy. Factors that influence the results of treatment. Arch Surg..

[227] Maddah G, Rajabi Mashhadi MT, Parvizi Mashhadi M, Nooghabi MJ, Hassanpour M, Abdollahi A (2017). Iatrogenic injuries of the extrahepatic biliary system. J Surg Res..

[228] Marino MV, Mirabella A, Guarrasi D, Lupo M, Komorowski AL (2019). Robotic-assisted repair of iatrogenic common bile duct injury after laparoscopic cholecystectomy: surgical technique and outcomes. Int J Med Robot..

[229] Huang Q, Yao HH, Shao F, Wang C, Hu YG, Hu S, Qiu LJ (2014). Analysis of risk factors for postoperative complication of repair of bile duct injury after laparoscopic cholecystectomy. Dig Dis Sci..

[230] Dominguez-Rosado I, Sanford DE, Liu J, Hawkins WG, Mercado MA (2016). Timing of surgical repair after bile duct injury impacts postoperative complications but not anastomotic patency. Ann Surg..

[231] Li J, Frilling A, Nadalin S, Paul A, Malago M, Broelsch CE (2008). Management of concomitant hepatic artery injury in patients with iatrogenic major bile duct injury after laparoscopic cholecystectomy. Br J Surg..

[232] Schmidt SC, Settmacher U, Langrehr JM, Neuhaus P (2004). Management and outcome of patients with combined bile duct and hepatic arterial injuries after laparoscopic cholecystectomy. Surgery..

[233] Ismael HN, Cox S, Cooper A, Narula N, Aloia T (2017). The morbidity and mortality of hepaticojejunostomies for complex bile duct injuries: a multi-institutional analysis of risk factors and outcomes using NSQIP. HPB (Oxford)..

[234] Post cholecystectomy bile duct injury: early, intermediate or late repair with hepaticojejunostomy - an E-AHPBA multi-center study*.* HPB (Oxford). 2019; 21: 1641-1647. 10.1016/j.hpb.2019.04.003. 10.1016/j.hpb.2019.04.003 31151812

[235] Sah DN, Bhandari RS (2020). Iatrogenic bile duct injury during cholecystectomy presenting after 11 years as a biliary stricture: a case report. J Med Case Rep..

[236] Sicklick JK, Camp MS, Lillemoe KD, Melton GB, Yeo CJ, Campbell KA, Talamini MA, Pitt HA, Coleman JA, Sauter PA, Cameron JL (2005). Surgical management of bile duct injuries sustained during laparoscopic cholecystectomy: perioperative results in 200 patients. Ann Surg..

[237] Walsh RM, Henderson JM, Vogt DP, Brown N (2007). Long-term outcome of biliary reconstruction for bile duct injuries from laparoscopic cholecystectomies. Surgery..

[238] Malla BR, Rajbhandari N, Karmacharya RM (2020). Management of bile duct injury following cholecystectomy. J Nepal Health Res Counc.

[239] Akaraviputh T, Boonnuch W, Lohsiriwat V, Methasate A, Chinswangwatanakul V, Lert-akayamanee N, Lohsiriwat D (2006). Long-term results of large diameter hepaticojejunostomy for treatment of bile duct injuries following cholecystectomy. J Med Assoc Thai..

[240] AbdelRafee A, El-Shobari M, Askar W, Sultan AM, El Nakeeb A (2015). Long-term follow-up of 120 patients after hepaticojejunostomy for treatment of post-cholecystectomy bile duct injuries: a retrospective cohort study. Int J Surg..

[241] Jajja, M.R., A. Laboe, S. Hashmi, S.O. Nadeem, B.A. Sayed, and J.M. Sarmiento, Standardizing diagnostic and surgical approach to management of bile duct injuries after cholecystectomy: long-term outcomes of patients treated at a high-volume HPB center*.* J Gastrointest Surg. 2021. 10.1007/s11605-021-04916-3. 10.1007/s11605-021-04916-3 33532980

[242] Barbier L, Souche R, Slim K, Ah-Soune P (2014). Long-term consequences of bile duct injury after cholecystectomy. J Visc Surg..

[243] Halle-Smith JM, Hodson J, Stevens LG, Dasari B, Marudanayagam R, Perera T, Sutcliffe RP, Muiesan P, Isaac J, Mirza DF, Roberts KJ (2020). A comprehensive evaluation of the long-term clinical and economic impact of minor bile duct injury. Surgery..

[244] Ozturk E, Can MF, Yagci G, Ersoz N, Ozerhan IH, Harlak A, Sahin M, Cetiner S, Tufan T (2009). Management and mid- to long-term results of early referred bile duct injuries during laparoscopic cholecystectomy. Hepatogastroenterology..

[245] Halbert C, Altieri MS, Yang J, Meng Z, Chen H, Talamini M, Pryor A, Parikh P, Telem DA (2016). Long-term outcomes of patients with common bile duct injury following laparoscopic cholecystectomy. Surg Endosc..

[246] Perera MT, Silva MA, Shah AJ, Hardstaff R, Bramhall SR, Issac J (2010). Risk factors for litigation following major transectional bile duct injury sustained at laparoscopic cholecystectomy. World J Surg..

[247] Cancer, C.P.a (2016). Improving patient care with Pan-Canadian synoptic-surgery reporting standards.

