# Supplementary figures and images for: 2020 WSES guidelines for the detection and management of bile duct injury during cholecystectomy
Source: World J Emerg Surg. 2021 Jun 10;16:30. doi: 10.1186/s13017-021-00369-w (PMC8190978; doi:10.1186/s13017-021-00369-w)

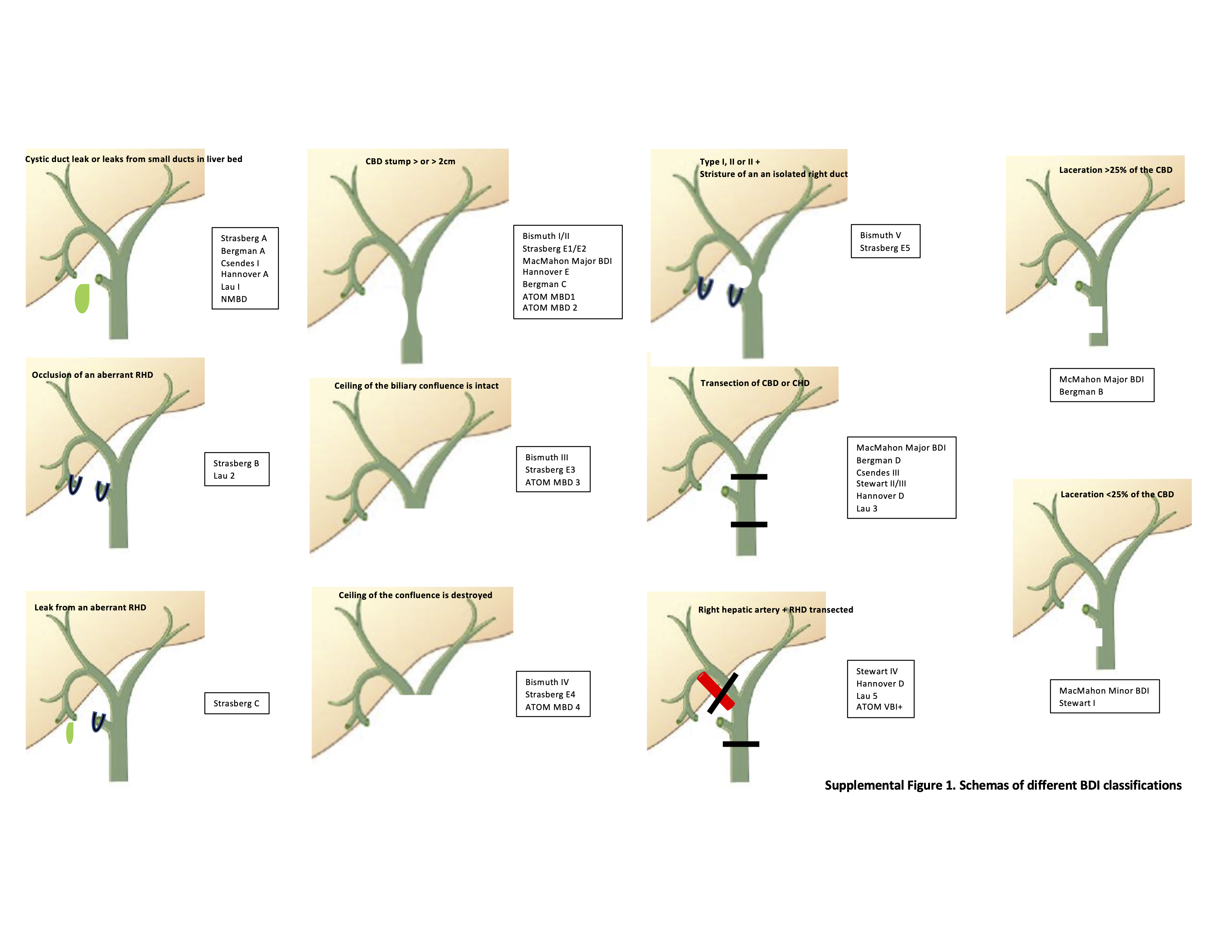

Supplement: Supplementary file 1 — Additional file 1. [file 13017_2021_369_MOESM1_ESM.tiff]

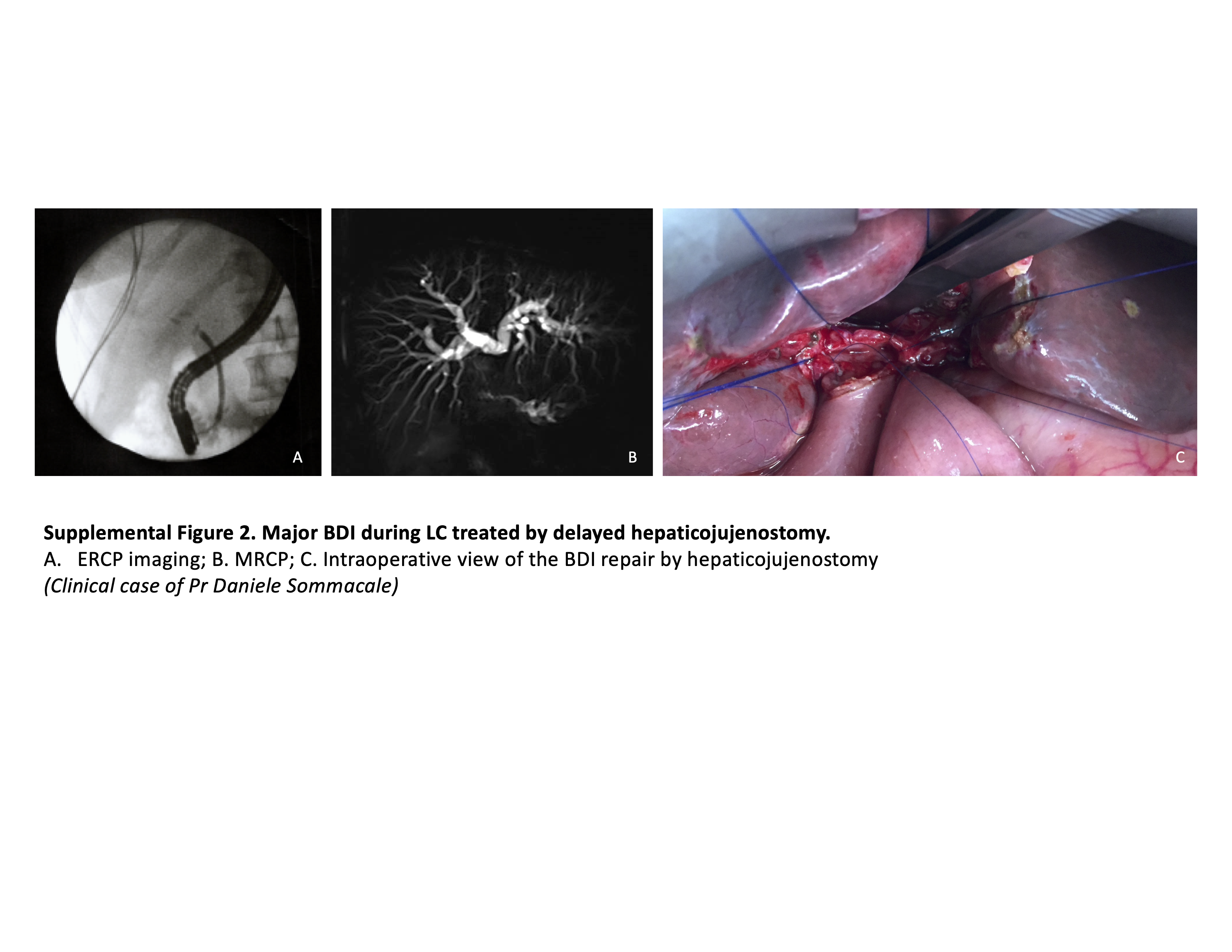

Supplement: Supplementary file 2 — Additional file 2. [file 13017_2021_369_MOESM2_ESM.tiff]
